# Male Meiosis as a Biomarker for Endo- to Ecodormancy Transition in Apricot

**DOI:** 10.3389/fpls.2022.842333

**Published:** 2022-04-07

**Authors:** Sara Herrera, Jorge Lora, Erica Fadón, Afif Hedhly, José Manuel Alonso, José I. Hormaza, Javier Rodrigo

**Affiliations:** ^1^Centro de Investigación y Tecnología Agroalimentaria de Aragón (CITA), Departamento de Ciencia Vegetal, Zaragoza, Spain; ^2^Instituto Agroalimentario de Aragón-IA2 (CITA-Universidad de Zaragoza), Zaragoza, Spain; ^3^Subtropical Fruit Crops Department, Instituto de Hortofruticultura Subtropical y Mediterránea La Mayora (IHSM La Mayora-CSIC-UMA), Málaga, Spain

**Keywords:** chilling requirements, heating requirements, *Prunus armeniaca*, dormancy, modeling, pollen development, dynamic model, flower buds

## Abstract

Dormancy is an adaptive strategy in plants to survive under unfavorable climatic conditions during winter. In temperate regions, most fruit trees need exposure to a certain period of low temperatures to overcome endodormancy. After endodormancy release, exposure to warm temperatures is needed to flower (ecodormancy). Chilling and heat requirements are genetically determined and, therefore, are specific for each species and cultivar. The lack of sufficient winter chilling can cause failures in flowering and fruiting, thereby compromising yield. Thus, the knowledge of the chilling and heat requirements is essential to optimize cultivar selection for different edaphoclimatic conditions. However, the lack of phenological or biological markers linked to the dormant and forcing periods makes it difficult to establish the end of endodormancy. This has led to indirect estimates that are usually not valid in different agroclimatic conditions. The increasing number of milder winters caused by climatic change and the continuous release of new cultivars emphasize the necessity of a proper biological marker linked to the endo- to ecodormancy transition for an accurate estimation of the agroclimatic requirements (AR) of each cultivar. In this work, male meiosis is evaluated as a biomarker to determine endodormancy release and to estimate both chilling and heat requirements in apricot. For this purpose, pollen development was characterized histochemically in 20 cultivars over 8 years, and the developmental stages were related to dormancy. Results were compared to three approaches that indirectly estimate the breaking of dormancy: an experimental methodology by evaluating bud growth in shoots collected periodically throughout the winter months and transferred to forcing chambers over 3 years, and two statistical approaches that relate seasonal temperatures and blooming dates in a series of 11–20 years by correlation and partial least square regression. The results disclose that male meiosis is a possible biomarker to determine the end of endodormancy and estimate AR in apricot.

## Introduction

Temperate woody perennials survive at low temperatures in winter by entering a dormant stage. This mechanism is characterized by the absence of visible growth in any plant structure containing a meristem and is associated with a slowdown of biological activity, a modification in hormonal balance, and extensive metabolic remodeling (Horvath et al., 2003; Rohde and Bhalerao, 2007).

However, dormancy is not just a survival strategy (Rohde and Bhalerao, 2007). Indeed, the temperature is a conditional factor in woody plants in temperate climates (Cooke et al., 2012; Ríos et al., 2014). Thus, chilling accumulation is required for proper flowering since the lack of winter chill during the winter period can cause physiological disorders such as bud burst delay and erratic and insufficient flowering negatively affecting yield (Viti et al., 2008; Campoy et al., 2011).

Although the need to accumulate chill for flowering and fruiting has been known for over 2 centuries (Knight, 1801), the biological basis behind remains elusive (Lloret et al., 2018; Goeckeritz and Hollender, 2021). However, the importance of chilling requirements (CR) in fruit trees is growing due to the increased release of new cultivars in many fruit tree crops, the expansion to warmer areas, and the lower winter chilling temperatures caused by climate change in many regions of the world (Luedeling et al., 2011).

Lang et al. (1987) proposed three different stages in dormancy: endodormancy, referred to when the regulation of dormancy is triggered by physiological factors; ecodormancy that refers to dormancy regulated by environmental factors; and paradormancy, which is used when growth inhibition arises from another part of the plant (e.g., apical dominance). The key factor to estimate agroclimatic requirements (AR; chilling and heat requirements) of particular cultivars is determining when the tree has overcome the phase of endodormancy and fulfilled its CR. Several approaches have been developed to estimate CR. For this purpose, the date of breaking of endodormancy has been experimentally determined by sequentially collecting shoots from dormant trees during the winter, exposing them to warm temperatures in controlled conditions, and quantifying chilling temperatures up to shoot collecting time when flower buds are considered capable to resume growth (Fadón et al., 2020a). Alternatively, the end of endodormancy has been determined by statistical correlations (Alonso et al., 2005) or partial least squares (PLS) regressions (Luedeling et al., 2013) with data of flowering dates and temperatures from long series of years. Both types of approaches are time-consuming, and in many cases, the results are not directly comparable under different climatic conditions (Fadón and Rodrigo, 2018). In the experimental approach, the use of cut shoots cannot entirely reflect the behavior of adult trees in field conditions, and several factors may affect the establishment of the date of endodormancy release and hence the estimations of CR (Fadón and Rodrigo, 2018). Likewise, statistical correlations require data from long series of years that are only available for a few sets of cultivars that exclude new cultivars, and the results may not fit equally well in different climates and latitudes (Dennis, 2003; Fadón et al., 2020a).

Several models have been used to quantify chilling and heating accumulation through hourly temperature data. The dynamic model has been reported as the most accurate model of chilling quantification (Ruiz et al., 2007; Luedeling and Brown, 2011; Campoy et al., 2012b,^2019^; Luedeling and Gassner, 2012). However, CR has also been calculated using Utah (Richardson et al., 1974) and Weinberger (1950) models to compare the results with those of the previous studies.

The dynamic model assumes that chilling accumulation is a two-step process, in which previous warm temperatures can counteract the effect of low temperatures during endodormancy. Initially, cold temperatures would promote the accumulation of an intermediate product. However, warm temperatures during this period of accumulation can stimulate the degradation of this precursor and reverse the process. Once the accumulation of this product reaches a critical concentration, it converts to a chill portion (CP), a stable factor, which cannot be destroyed, and promotes the break of the endodormancy (Fishman et al., 1987). The Utah model establishes temperature ranges that contribute differently to chilling accumulation, which is measured as chill units (CU) (Richardson et al., 1974). The chill accumulation takes place within a temperature range of 1.5–12.4°C (Richardson et al., 1974). Finally, the Weinberger model proposes that only temperatures below 7.2°C (45°F) are useful and have an effect in breaking the endodormancy (chilling hours, CH) (Weinberger, 1950).

Nowadays, CR in most cultivars is unknown, mainly due to the problems associated with the existing methods and the lack of a biological marker to determine the chilling fulfillment of a tree in field conditions (Fadón and Rodrigo, 2018). In previous works in apricot, anther differentiation has been related to winter dormancy since it sets a boundary between sporogenous tissue and pollen grain formation stages (Julian et al., 2011). The stamens remain in this anatomically quiescent stage during endodormancy to resume growth and complete the development of pollen grains in ecodormancy (Rodrigo and Herrero, 2002). However, male meiosis takes place once CR has been fulfilled but flower buds do not show external signs of development (Bartolini et al., 2006; Julian et al., 2014).

On the other hand, pollen development is a well-characterized and highly conserved process in angiosperms. Prior to meiosis, microspore mother cells become isolated by a wall with the deposition of a callose layer (McCormick, 2004). Callose is a linear homopolymer widely found in higher plants with important roles in plant growth and development (Chen and Kim, 2009). During pollen development, callose may act as a physical molecular filter involved in stress responses (Dong et al., 2005; Blackmore et al., 2007).

In this work, we evaluate male meiosis as a biological marker of breaking dormancy for estimating AR by histochemical detection of callose deposition around microspore mother cells. For this purpose, chilling and heating requirements of a group of 20 apricot cultivars have been estimated by quantifying chill accumulation until the date of male meiosis and heat accumulation from male meiosis to flowering for over 4 years. To validate the results with those obtained with other existing methods, endodormancy breaking has also been experimentally estimated by studying cut shoots under forcing conditions, by two statistical models that estimate the correlation between blooming dates and seasonal temperatures and the determination of the daily influence of chill accumulation in blooming dates by PLS regression.

## Materials and Methods

### Plant Material

Plant material was obtained from an experimental orchard located at the Centro de Investigación y Tecnología Agroalimentaria de Aragón (CITA) in Zaragoza (Spain) at 41°44′30″ N, 0°47′00″ W and 220 m altitude. Twenty apricot cultivars with different flowering dates were selected. Phenological observations of flower buds were carried out two times a week in each cultivar from bud break to flowering. Blooming dates were recorded according to Baggiolini stages, considering full flowering (F50) when 50% of flower buds were in stage F (Baggiolini, 1952) that corresponds to stage 65 (full bloom) in the BBCH scale (Meier, 2001; Pérez-Pastor et al., 2004). Daily temperatures during 1998–2021 were acquired from a meteorological station close to the orchard.

### Determination of Endodormancy Breaking

Four different procedures were carried out to estimate the chilling and heating requirements for flowering.

#### Forcing Test: Cutting Shoots Under Forcing Conditions

Flower bud growth was evaluated in response to warm conditions after exposure to chill in the field. Four shoots of each cultivar were collected weekly from November to March over 3 years (2016–2017, 2017–2018, and 2019–2020). The shoots with lengths of around 40 cm were placed in a growth chamber with controlled conditions and maintained on a wet florist foam. The end of the shoot was cut underwater to avoid cavitation and the water was renovated periodically. The shoots were maintained at 22 ± 1°C with a 12-h light photoperiod for 8 days. To determine the end of endodormancy, 10 flower buds were randomly picked and weighed on the first and last day in the growth chamber. Endodormancy was considered overcome when a significant bud weight increase (30%) was observed after the period under controlled conditions (Brown and Kotob, 1957; Tabuenca, 1964; Ruiz et al., 2007). The end of endodormancy for each cultivar was established for each year.

#### Correlation of Long Term Records of Blooming Dates and Daily Winter Temperatures

A statistical model based on the temperature effects on blooming dates in 13 years was carried out for each cultivar. Two matrixes were defined to determine the end of endodormancy. One of them was based on blooming dates composed of two axes: years and genotypes. The other one was defined by the number of days from October 1 (day 1) to April 30 (day 212) and the average of the mean daily temperature measured at 15 day periods (Alonso et al., 2005). The vector obtained on each day was correlated to the corresponding vector of the blooming dates matrix and the Pearson correlation coefficient (PCC) was calculated. The endodormancy to ecodormancy period was established when no significant correlation coefficients were found, i.e., from the last day significantly positive (PCC = 0.553) to the first day significantly negative (PCC = −0.553). The analysis was based on a dataset of 13 years with the Student’s *t*-test for 11 df and a two-tailed test at α = 0.05. The end of endodormancy was the mean date of the transition period in each cultivar.

#### Partial Least Squares Regression Analysis of Daily Chill, Heat Accumulation, and Blooming Dates

Partial least squares regression analysis was carried out to establish chilling and heating phases (Luedeling et al., 2013). Blooming dates for a period between 11 and 18 years, depending on the cultivar, were correlated with daily chill and heat accumulation. Chill accumulation was measured as chill portions of the dynamic model whereas growing degree hours (GDH) were used to measure heat accumulation (Fadón et al., 2021).

The PLS analysis calculates model coefficients that indicate the effect of the chill portions in the blooming dates. Negative coefficients have a blooming-advancing effect whereas positive coefficients are related to high temperatures during the chilling period that results in a delay in blooming. The absolute value of the model coefficients indicates the strength of the effect. The variable-importance-in-the-projection (VIP) values were used to define the chilling and heating phases. Values greater than VIP = 0.8 indicate significant importance of model coefficients (Luedeling and Gassner, 2012; Luedeling et al., 2013; Fadón et al., 2021). A single date for the beginning and the end of the chilling and forcing periods was defined per cultivar.

#### Male Meiosis Characterization

Flower buds were sampled weekly during winter, from November to February, for 8 years. The buds were fixed in ethanol (95%)/acetic acid (3:1, v/v) for 24 h and conserved at 4°C in ethanol 75% (Williams et al., 1999). To determine the stage of the development of pollen, anthers were removed from fixed buds with the help of a scalpel and mounted by the squash technique adding 0.1% aniline blue in 0.1 N K_3_PO_4_ to stain callose (Currier, 1957). Microscopic preparations were observed under a Leica DM2500 microscope (Cambridge, United Kingdom) with UV epifluorescence using 340–380 bandpass and 425 longpass filters. The formation of a callose wall around the microspore mother cells was established as the date when the CR was fulfilled (Julian et al., 2014). The end of the endodormancy was determined per year in each cultivar.

### Quantification of Chilling and Heat Requirements

The Weinberg, Utah, and dynamic models were used to quantify CR. The amount of chill required to overcome endodormancy was calculated from the initial date of chilling accumulation until the date of endodormancy release established by the different methods. The day considered as the beginning of chilling accumulation varied between the models. According to Weinberger (1950), chilling hours are the number of hours below 7°C from the time in autumn when chill hours start to occur. However, in the Utah model, the day when the temperatures produced the maximum negative effect is considered as the starting date of the accumulation of chill units (Richardson et al., 1974). In the dynamic model, we established 1st September as the starting date of accumulation of portion units (Fishman et al., 1987). That date fits well with the climatic conditions in Zaragoza, where usually no chilling occurs before it.

In the two experimental methods (forcing test and male meiosis), CR was calculated per year and the mean value was established per cultivar. Since the statistical approaches determined a single date for the end of the endodormancy, a unique chilling requirement value was obtained per cultivar.

Heat requirements were expressed as the accumulation of GDH from endodormancy breaking to flowering. The GDH corresponds to an accumulation of hours above 4.5°C from the date established as the end of the endodormancy to the date when 50% of the flowers were open (F50) (Richardson et al., 1974).

### Statistical Analyses

All the analyses were performed in the R programming environment v. 4.1.0 (R Core Team, 2022). The PLS analysis was carried out using the chillR package (version 0.70.24) (Luedeling, 2019).

The coefficient of variation was used as the indicator of quality within years for each method. To compare the results obtained from the four different methods and evaluate the potential of bias between them, we carried out a difference plot analysis using the mean chill portions values per cultivar through the Bland–Altman method (Bland and Altman, 1986, 1999) and the MethComp (Carstensen et al., 2013) packages for R. To assess the level of agreement between a pair of measurement methods, two-by-two analyses were performed to establish a two-sided 95% confidence interval (Shieh, 2018) and whenever 95% of the scatter points fell within that interval both methods were considered to be in agreement.

## Results

**FIGURE 1:**
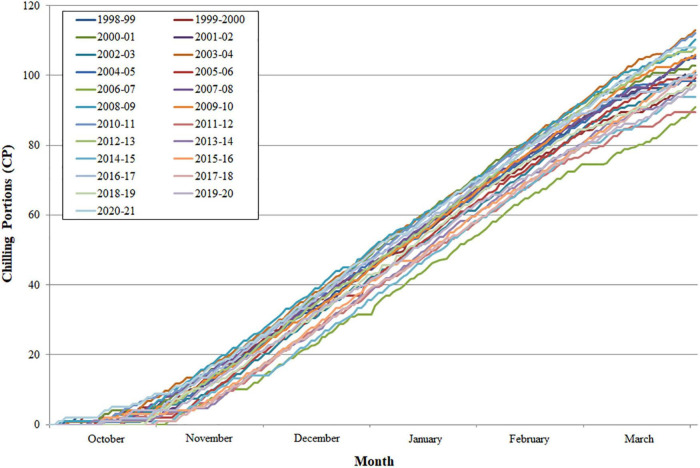
Mean daily chill portion accumulation per year for the period 1998–2021 at Zaragoza (Spain).

Chill accumulation started in October in all the years analyzed and increased exponentially during the winter months. However, significant differences were observed between years, ranging from 89.4 chilling portions (CP) (2011–2012) to 112.9 CP (2003–2004) accumulated at the end of March (Figure 1).

### Establishment of Endodormancy Breaking by Cut Shoots Subject to Forcing Conditions

**FIGURE 2:**
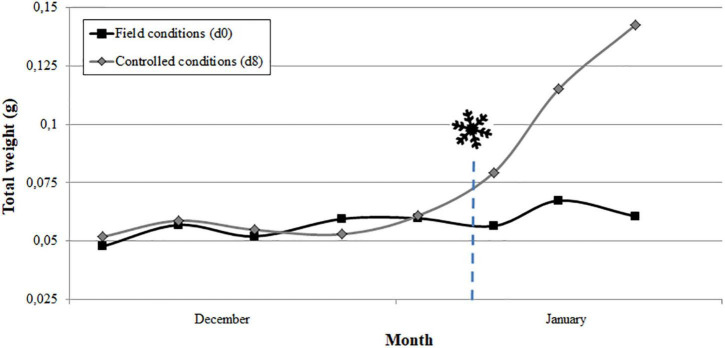
Estimation of breaking of endodormancy date (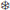) in the apricot cultivar “Pandora” in 2019 by the Forcing test. Flower bud weight in field conditions and after 8 days in the growth chamber. Chilling requirements were considered fulfilled when the increase in the weight of the flower buds in the growth chamber was 30% higher than in field conditions (*n* = 10).

For each cultivar and year, the comparison of the evolution of flower bud growth under field and controlled conditions allowed determining the endodormancy breaking date when the flower buds increased their weight by 30% after 8 days in the growth chamber (Supplementary Table 1). As an example, the date of endodormancy breaking for “Pandora” was 6 January 2019 (Figure 2). The average date of endodormancy breaking for the 3 years analyzed ranged between December 27 and February 8 between cultivars (Table 1).

**TABLE 1:** Dates of endodormancy and chilling requirements of 20 apricot cultivars determined according to four methods (forcing test, correlation, PLS, and male meiosis).

	Chilling requirements (Chilling portions)											
	Forcing test			Correlation model			PLS regression			Male meiosis		
	Endodormancy breaking	Value ± SD	cv	Endodormancy breaking	Value ± SD	cv	Endodormancy breaking	Value ± SD	cv	Endodormancy breaking	Value ± SD	cv
Berdejo	06 Jan	47.2 ± 4.5	9.6	18 Jan	57.0 ± 4.9	8.7	14 Jan	54.2 ± 4.6	8.5	26 Jan	61.9 ± 5.1	8.3
Canino	27 Dec	40.2 ± 6.1	15.1	18 Jan	57.0 ± 4.9	8.7	15 Jan	54.8 ± 4.7	8.6	14 Jan	53.2 ± 6.2	11.6
Corbato	07 Jan	47.5 ± 1.5	3.1	16 Jan	55.7 ± 4.8	8.7	15 Jan	54.9 ± 4.5	8.3	23 Jan	59.7 ± 4.6	7.6
Goldrich	29 Dec	42.0 ± 7.7	18.2	17 Jan	56.4 ± 4.8	8.6	13 Jan	53.6 ± 3.9	7.3	21 Jan	58.3 ± 5.5	9.4
Gönci Magyar	25 Jan	60.2 ± 5.1	8.4	04 Feb	69.1 ± 5.0	7.2	12 Feb	74.8 ± 5.1	6.8	02 Feb	66.8 ± 5.2	7.7
Harcot	03 Jan	45.0 ± 2.5	5.6	15 Jan	54.8 ± 4.9	8.9	13 Jan	53.3 ± 4.1	10.7	23 Jan	59.8 ± 5.2	8.7
Henderson	05 Feb	69.1 ± 5.2	7.6	04 Feb	69.1 ± 5.0	7.2	13 Jan	53.6 ± 3.9	7.3	07 Feb	70.9 ± 10.4	14.6
Luizet	07 Jan	48.0 ± 4.0	8.3	19 Jan	58.0 ± 4.9	8.5	28 Jan	64.0 ± 4.5	7.1	25 Jan	61.3 ± 4.8	7.9
Mitger	07 Jan	47.5 ± 1.5	3.1	16 Jan	55.7 ± 4.8	8.7	29 Dec	43.2 ± 3.9	9.1	21 Jan	58.0 ± 5.4	9.3
Moniqui 1006	04 Jan	45.8 ± 3.5	7.6	15 Jan	54.8 ± 4.9	8.9	17 Dec	33.9 ± 4.6	13.6	23 Jan	59.6 ± 5.3	8.9
Moniqui 2113	10 Jan	49.8 ± 2.4	4.9	16 Jan	55.7 ± 4.8	8.7	14 Jan	55.0 ± 4.3	14.0	26 Jan	61.7 ± 5.9	9.6
Muñoz	05 Jan	46.5 ± 3.8	8.2	18 Jan	57.0 ± 4.9	8.7	21 Jan	58.6 ± 4.4	14.0	22 Jan	59.1 ± 4.8	8.1
Pandora	04 Jan	45.8 ± 3.5	7.6	18 Jan	57.0 ± 4.9	8.7	13 Jan	53.6 ± 3.9	7.3	20 Jan	60.7 ± 9.6	15.8
Paviot	06 Jan	47.2 ± 4.5	9.6	18 Jan	57.0 ± 4.9	8.7	14 Jan	54.3 ± 4.4	8.1	25 Jan	61.6 ± 5.0	8.1
Pepito del Rubio	27 Dec	41.2 ± 4.1	10.0	16 Jan	55.7 ± 4.8	8.7	13 Jan	53.3 ± 4.3	13.2	22 Jan	58.9 ± 5.1	8.7
Stark Early Orange	25 Jan	60.4 ± 2.6	4.3	06 Feb	70.7 ± 5.0	7.1	14 Feb	76.7 ± 4.2	5.5	08 Feb	71.4 ± 6.4	9.0
Stella	08 Feb	69.9 ± 6.7	9.5	22 Feb	81.7 ± 5.3	6.4	09 Feb	72.9 ± 4.5	6.1	01 Mar	84.8 ± 7.5	8.8
Sun Glo	15 Jan	53.9 ± 3.5	6.4	20 Jan	58.6 ± 4.9	8.4	13 Jan	53.6 ± 3.9	7.3	01 Feb	66.3 ± 7.0	10.6
Tadeo	09 Jan	49.1 ± 2.4	5.0	10 Feb	73.5 ± 5.0	6.8	13 Jan	53.6 ± 3.9	7.3	24 Jan	60.5 ± 5.3	8.7
Veecot	09 Jan	48.9 ± 1.7	3.5	19 Jan	58.0 ± 4.9	8.5	14 Jan	54.6 ± 4.0	7.3	16 Jan	54.5 ± 5.4	9.8

*Values are in chill portions.*

*SD, standard deviation; cv, coefficient of variation.*

### Determination of the Transition From Endodormancy to Ecodormancy by Correlation Modeling

**FIGURE 3:**
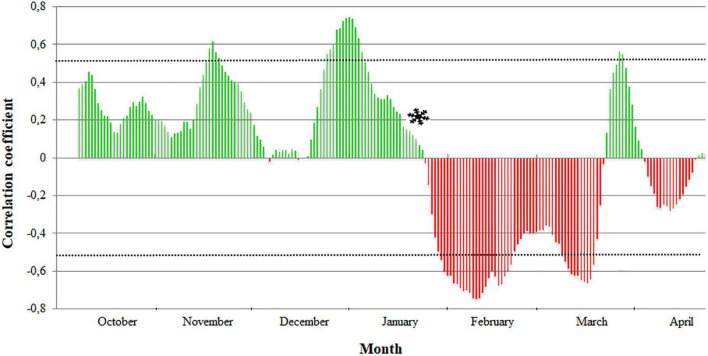
Establishment of endodormancy breaking date (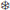) in the apricot cultivar “Pandora” by analyzing the evolution of the Pearson correlation coefficients between the full flowering dates and the winter temperatures of periods of 15 days. Discontinuous lines delimit the significant values (0.553, –0.553). Green and red scales show positive and negative coefficients, respectively.

For each cultivar, the correlation coefficients between the full flowering dates and the winter temperatures were obtained using the mean daily temperature in periods of 15 days over 13 years (Supplementary Figure 1). The transition from endodormancy to ecodormancy was established when the last significant positive correlation coefficient was obtained to the first significant negative correlation coefficient. The dates of endodormancy breaking ranged from January 15 to February 22 (Table 1 and Supplementary Table 2). As an example, the endodormancy breaking date in “Pandora” was estimated on January 18 (Figure 3).

### Determination of Chilling and Forcing Periods by Partial Least Squares Regression

**FIGURE 4:**
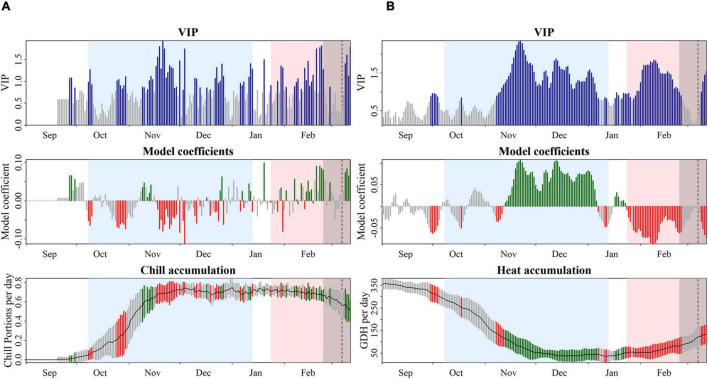
Model coefficients of a partial least squares regression model of daily accumulation of winter chill [**(A)** quantified in Chill Portions] and heat [**(B)** quantified in growing degree hours] and bloom dates of the apricot cultivar “Pandora.” Top panels show the variable importance in the projection (VIP), with the blue bars values above 0.8 indicating the threshold for variable importance. Middle panels show the model coefficients of the centered and scaled data. The chilling period is colored in blue and the heating period is colored in red. The bottom panels show mean temperatures (black line) and their standard deviation (gray areas). In the middle and bottom panels, red and green scales show negative and positive coefficients, respectively.

Chilling and forcing phases for each cultivar were defined based on the results of the PLS analysis with daily chill accumulation quantified in CP. High daily chill accumulation rates during the chilling phase generally indicated a correlation with early blooming dates. The beginning and the end of the chilling periods extended from negative significant coefficients occurring in September–October, around endodormancy establishment, to negative significant coefficients in December–January, indicating the end of endodormancy. As an example, in “Pandora,” the chilling period was considered from 8th October to 13th January, when a clear trend of negative model coefficients, as well as predominantly significant VIP scores, was shown (Figure 4A). The starting dates in all the cultivars took place in a short period, from 29th September to 9th October (Supplementary Table 3). However, the dates of endodormancy breaking were highly variable, ranging from 17th December to 14th February (Table 1).

The forcing periods were derived from the PLS analysis based on daily heat accumulation. Negative model coefficients and high VIP scores indicated that high daily heat accumulation during this period had a strong effect on bloom-advancing dates. Forcing periods were highly variable between cultivars, lasting between 30 and 71 days. The start date of ecodormancy ranged from early-January to late-February (Table 1). As an example, the forcing phase in “Pandora” was estimated from 24th January to 11th March (Figure 4B).

For all cultivars, the forcing period did not follow directly after the chilling period, but it was preceded by a transition phase during which the coefficients were neither clearly negative nor clearly positive, and they were not considered by the VIP analysis. This transition period occurred between January and February and ranged from 3 to 20 days depending on the cultivars (Supplementary Figure 2).

### Anther Development and Dormancy Phases

**FIGURE 5:**
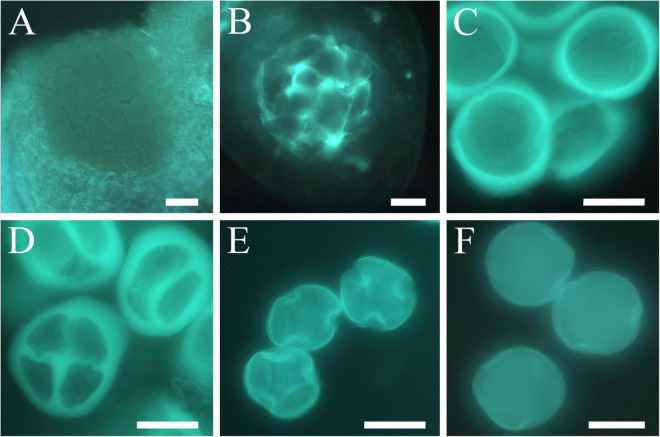
Anther development and microsporogenesis in apricot. **(A)** Sporogenous tissue. **(B)** Appearance of callose indicating the onset of microspore mother cell differentiation. **(C)** Early-stage microspore mother cells surrounded by callose layering. **(D)** Tetrads. **(E)** Microspores released. **(F)** Mature pollen grain. Scale bars: 20 μm.

Sporogenous tissue (Figure 5A) was observed in the anthers of all the cultivars during several months of the endodormancy phase when no signs of development were observed. The deposition of callose around the sporogenous tissue cells was the first sign of reactivation of anther development (Figure 5B). The onset of meiosis began with the accumulation of a callose layer around the microspore mother cells that became isolated (Figure 5C). About a week later, two meiotic divisions occurred rapidly forming the tetrads, a stage of four haploid cells surrounded by callose (Figure 5D). After meiosis, the callose wall dissolved, closely followed by the release of the young microspores (Figure 5E), which developed into mature pollen grains before anthesis (Figure 5F).

**FIGURE 6:**
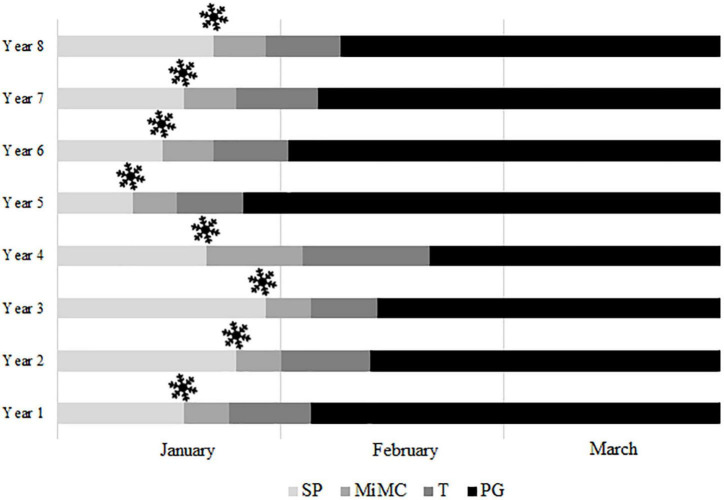
Anther development from endodormancy to flowering in the apricot cultivar “Pandora” over eight years. SP, sporogenous tissue; MiMC, microspore mother cells; T, tetrads; PG, pollen grains; 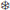, estimation of endodormancy breaking date.

The average dates of breaking of endodormancy, considering the first appearance of callose surrounding the microspore mother cells, were established in all the cultivars from 14th January to 1st March (Table 1), although differences were observed between years for each cultivar (Supplementary Table 4). As an example, the onset of meiosis in “Pandora” ranged between January 12 and January 30 (Figure 6).

In all the cultivars, the end of the endodormancy took place earlier according to the forcing test than in PLS regression, correlation model, and male meiosis, respectively.

### Chilling Requirements

Once the end of the endodormancy was established for each method, the CR was estimated by quantifying the CP accumulated until that date. The lowest values were found in PLS regression (30.9–76.7 CP) which showed a broader range than the other methods [forcing test (40.2–69.9 CP), correlation model (53.2–84.2 CP), and male meiosis (54.8–81.7 CP)]. The cultivars with higher CR (“Gönci Magyar,” “Stark Early Orange,” and “Stella”) were the same in all methods. However, the cultivars with lower CR showed some differences depending on the type of method, experimental or statistical. Both correlation and PLS methods shared a group of lower chilling cultivars (“Harcot,” “Mitger,” “Moniqui 1006,” “Moniqui 2113,” and “Pepito del Rubio”), in addition to “Muñoz” in the PLS method, and “Corbato” in the correlation method. Both experimental methods (forcing test and male meiosis) reported the same lower CR cultivars (“Canino,” “Goldrich,” and “Pepito del Rubio”) but higher values were observed in the male meiosis method.

**FIGURE 7:**
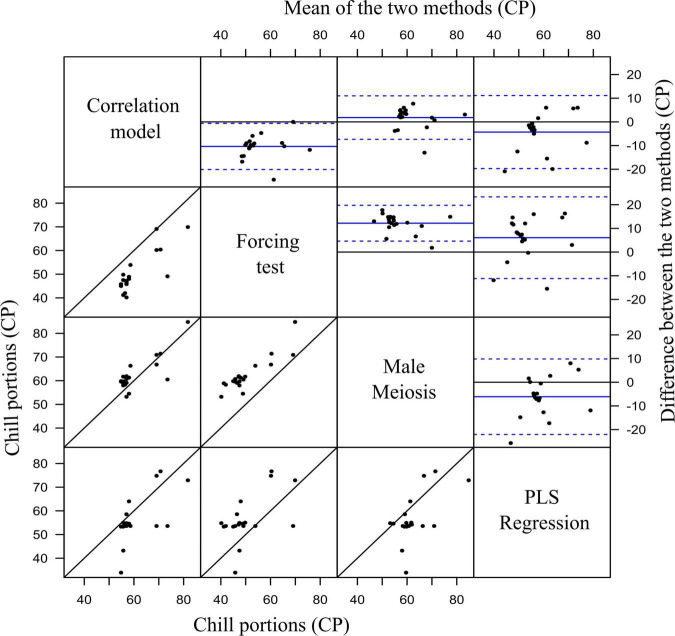
Graphical methods for assessing variability and agreement in paired measurements of chill portion values obtained from four different methods. Scatter diagrams (lower-left grid) show the set of paired values in each pairwise comparison. Bland-Altman plots (upper-right grid) of the difference in chill portions of each value-pair against the mean of each value-pair. Blue horizontal lines show the mean difference (solid lines) and the superior and inferior limits of agreement (± 1.96⋅SD), respectively (dotted lines).

The Bland–Altman analysis was used to look for potential bias between the four methods by plotting the difference in chill portions per cultivar against the mean in pairwise comparisons (Figure 7) and assessing their agreement level. Most of the scatter points on the Bland–Altman plots were uniformly scattered between the limits of agreement in all the comparisons, suggesting the absence of bias. About 95% of cultivars would have a difference in chill portions, as measured by any two different methods, within the limits of agreement, suggesting good agreements. The 20 cultivars were classified into three groups according to their CR (Table 2).

**TABLE 2:** Classification of 20 apricot cultivars according to their chilling requirements (Chill portions) determined by the male meiosis method.

High chilling requirements (≥66 CP)	Henderson, Gönci Magyar, Stark Early Orange, Stella, Sun Glo
Medium chilling requirements (56–65 CP)	Berdejo, Corbato, Goldrich, Harcot, Luizet, Mitger, Moniqui 1006, Moniqui 2113, Muñoz, Pandora, Paviot, Pepito del Rubio, Tadeo
Low-medium chilling requirements (50–55 CP)	Canino, Veecot

### Heat Requirements

The heat requirements of cultivars showed differences according to the method used. The highest values were obtained with the forcing test (2,866–5,936 GDH) whereas the PLS method showed the lowest values (3,448–5,468 GDH). In the correlation model, the heating requirements values were uniform between cultivars ranging from 4,022 to 4,953 GDH, except for “Tadeo,” which showed the lowest value (3,000 GDH) (Table 3). The values of heat requirements displayed higher coefficients of variability than those obtained for CR. The 20 cultivars were classified into three groups according to their heat requirements (Table 4).

**TABLE 3:** Heat requirements from the end of endodormancy to full flowering of 20 apricot cultivars determined according to four methods (forcing test, correlation model, PLS, and male meiosis methods).

	Heat requirements (Growing Degree Hours)							
	Forcing test		Correlation model		PLS regression		Male meiosis	
	Value ± SD	cv	Value ± SD	cv	Value ± SD	cv	Value ± SD	cv
Berdejo	5,278 ± 706	13.4	4,917 ± 882	17.9	3,905 ± 948	24.3	4,170 ± 498	11.9
Canino	5,026 ± 906	18.0	4,023 ± 784	19.5	3,634 ± 905	24.9	4,212 ± 765	18.2
Corbato	4,814 ± 263	5.5	4,520 ± 864	19.1	3,640 ± 870	23.9	3,900 ± 440	11.3
Goldrich	4,749 ± 1,146	24.1	4,086 ± 791	19.4	4,004 ± 976	24.3	3,712 ± 343	9.2
Gönci Magyar	4,288 ± 78	1.8	4,861 ± 887	18.2	3,448 ± 779	22.6	4,027 ± 513	12.7
Harcot	4,767 ± 470	9.9	4,689 ± 873	18.6	4,209 ± 937	22.3	3,998 ± 402	10.1
Henderson	2,866 ± 666	23.2	4,441 ± 837	18.8	4,604 ± 1,083	23.5	4,159 ± 968	23.3
Luizet	5,076 ± 407	8.0	4,732 ± 858	18.1	4,004 ± 906	22.6	4,120 ± 437	10.6
Mitger	4,777 ± 599	12.5	4,638 ± 860	18.5	5,326 ± 951	17.8	4,084 ± 467	11.4
Moniqui 1006	4,921 ± 399	8.1	4,821 ± 882	18.3	4,811 ± 921	19.1	4,045 ± 241	6.0
Moniqui 2113	5,144 ± 538	10.5	4,902 ± 884	18.0	5,305 ± 953	18.0	4,196 ± 542	12.9
Muñoz	5,170 ± 799	15.5	4,650 ± 845	18.2	3,934 ± 881	22.4	4,185 ± 527	12.6
Pandora	4,924 ± 993	20.2	4,141 ± 792	19.1	3,723 ± 926	24.9	3,863 ± 498	12.9
Paviot	5,119 ± 551	10.8	4,781 ± 860	18.0	3,721 ± 886	23.8	4,135 ± 411	9.9
Pepito del Rubio	5,296 ± 646	12.2	4,638 ± 860	18.5	3,852 ± 894	23.2	4,019 ± 312	7.8
Stark Early Orange	4,152 ± 76	1.8	4,123 ± 758	18.4	3,607 ± 819	22.7	4,053 ± 1,220	30.1
Stella	5,936 ± 191	3.2	4,511 ± 638	14.1	5,468 ± 847	15.5	3,879 ± 746	19.2
Sun Glo	4,915 ± 733	14.9	4,953 ± 944	19.1	5,054 ± 1,174	23.2	3,789 ± 472	12.5
Tadeo	4,742 ± 239	5.1	3,000 ± 514	17.1	4,604 ± 1,083	23.5	4,126 ± 576	14.0
Veecot	4,564 ± 700	15.3	4,091 ± 789	19.3	4,660 ± 1,093	23.5	4,284 ± 898	21.0

*Values are in growing degree hours.*

*SD, standard deviation; cv, coefficient of variation.*

**TABLE 4:** Classification of 20 apricot cultivars according to their heat requirements (growing degree hours) determined by the male meiosis method.

High heating requirements (≥4,101 GDH)	Berdejo, Canino, Henderson, Luizet, Moniqui 2113, Muñoz, Paviot, Tadeo, Veecot
Medium heating requirements (3,901–4,100 GDH)	Gönci Magyar, Harcot, Mitger, Moniqui 1006, Pepito del Rubio, Stark Early Orange
Low heating requirements (≤3,900 GDH)	Goldrich, Corbato, Pandora, Stella, Sun Glo

## Discussion

In this work, we have validated male meiosis as a biomarker for the determination of dormancy breaking by comparing the dormancy breaking dates calculated with three other common methodologies in 20 apricot cultivars. The results support our initial hypothesis of male meiosis as a suitable biomarker for endo- to ecodormancy transition.

The AR has been determined in 20 apricot cultivars, for ten of which (“Berdejo,” “Henderson,” “Mitger,” “Muñoz,” “Pandora,” “Pepito del Rubio,” “Stella,” “Sun Glo,” “Tadeo,” and “Veecot”) no previous data were available. This is the first time that a biomarker (male meiosis) and two statistical methods (statistical correlation and PLS regression) have been used to estimate chilling and heat requirements in apricot since all the previous data have been obtained with the experimental method of transferring shoots into a growth chamber sequentially during winter.

The CR reported herein ranged from 30.9 to 84.8 CP (Dynamic model), 470–1,681 CU (Utah model), and 410–1,437 CH (Weinberger model), which are in line with the results previously reported in this species (reviewed in Fadón et al., 2020a). Our estimate of the CR for “Canino” (875.4 CH *vs*. 750–878 CH), “Luizet” (1,016.3 CH *vs.* 1,058–1,150 CH), “Moniqui” (983.6 and 1,036.4 CH *vs.*, 799–1,057 CH), and “Paviot” (1,023.7 CH *vs*. 995–1,296 CH) by using male meiosis as a biomarker of endodormancy breaking was similar to what has been previously determined at the same location (Tabuenca, 1964, 1968, 1979) by using the forcing test (Supplementary Table 4). Three cultivars, “Canino,” “Harcot,” and “Goldrich” were previously studied in different regions, Tuscany (Italy) (Garcia et al., 1999; Viti et al., 2010) and South Africa (Campoy et al., 2012a) compared to Murcia (Spain). Although significant differences were reported between locations, our results under forcing conditions were similar to those reported in Murcia (Spain). In addition, our results showed similar CR values for some cultivars evaluated with the forcing test previously in other regions, such as “Moniqui” in Italy (921.7 and 1,003.1 CU *vs.* 930–1,140 CU; Guerriero et al., 2006), “Gönci Magyar” in Serbia (1,200.8 CU *vs.* 1,122–1,310 CU; Ruml et al., 2018), and “Goldrich” in Italy and Serbia (854.0 CU *vs*. 950–1,030 CU and 834–846 CU, respectively; Guerriero et al., 2006; Ruml et al., 2018; Supplementary Table 1).

The four methods showed the same group of cultivars with higher CR, and similar values were obtained between methods for each cultivar. However, some differences between methods were found for the cultivars with lower CR, “Canino,” “Golrich,” and “Pepito del Rubio” in both experimental methods, and “Harcot,” “Mitger,” “Moniqui 1006,” “Moniqui 2113,” and “Pepito del Rubio” in both statistical methods. Differences between experimental and statistical methods could be due to the different number of years used, 11–18 for statistical methods and 3–8 for experimental methods. Previous studies in apricot using the forcing test have reported differences in CR not only between years, but also between locations, which may be due to a small number of years of experiments (Garcia et al., 1999; Viti et al., 2010; Campoy et al., 2012a).

The classification of the cultivars obtained according to their CR was similar to that obtained by other authors (Ruiz et al., 2007; Campoy et al., 2012a). “Canino” has been considered a medium chill cultivar in previous works (Campoy et al., 2012a), whereas our results showed lower chill portions accumulation in “Canino” and “Veecot” than those in the other cultivars. Thus, we propose an intermediate group that includes cultivars with low-medium CR.

Differences in CR between methods affected heating requirements, showing a significant negative correlation as reported in previous work (Ruiz et al., 2007). Lower values of heat accumulation were found with the male meiosis method whereas higher values were obtained with the forcing test. The heat requirements reported for “Canino” (5,026 GDH) and “Harcot” (4,767 GDH) are in line with those reported in previous studies in Spain [5,418–6,029 GDH (Garcia et al., 1999; Campoy et al., 2012a)]. Our results showed higher heat accumulation for “Goldrich,” “Moniqui,” and “Gönci Magyar” than in other countries. As an example, for “Goldrich,” 4,479 GDH vs. 3,175–3,432 GDH in Serbia (Ruml et al., 2018) and 3,950 or 485–913 GDH in Italy (Guerriero et al., 2006; Viti et al., 2010).

Differences between methods as well as those in previous reports may also be due to the specific limitations of each method. The determination of endodormancy breaking by monitoring shoots under forcing conditions is the only experimental approach currently available and the most widely used to estimate the AR of fruit tree cultivars for more than 60 years (Brown and Kotob, 1957; Fadón et al., 2020a). However, the variability of the factors affecting the experimental design has often led to inconsistencies in the results previously obtained. In apricot, as in other species, the frequency of sampling of shoots during winter may vary between studies, as well as the temperature and photoperiod conditions in the growth chamber (Tabuenca, 1968, 1979; Andrés and Durán, 1999; Valentini et al., 2004; Ruiz et al., 2007; Bartolini et al., 2020). Furthermore, the determination of the endodormancy breaking date through the evaluation of bud growth can be carried out after different periods in the chamber. Several criteria are also used, such as significant increases in fresh (Brown and Kotob, 1957) or dry weight (Tabuenca, 1968), as well as changes in bud phenology (Bennett, 1949). Moreover, most studies evaluate flower buds, but some use vegetative buds (Fernandez et al., 2019). The lack of standardization has made most of the results not applicable to other regions with different climatic conditions (Campoy et al., 2019; Fadón et al., 2020a).

The statistical approaches estimate the date of chilling fulfillment based on a long series of phenological observations. Both correlations of winter temperatures with flowering dates and the PLS analysis have been widely used for the determination of AR in sweet cherry (Luedeling et al., 2013; Fadón et al., 2021), almond (Alonso et al., 2005; Benmoussa et al., 2017a; Díez-Palet et al., 2019), pistachio (Benmoussa et al., 2017b), and apple (Díez-Palet et al., 2019). In our work, results from both statistical methods have shown results similar to those from experimental methods. However, some works indicate the need for about 20 years of flowering date records to obtain consistent results (Luedeling and Gassner, 2012; Luedeling et al., 2013; Fadón et al., 2021), which is one of the main pitfalls of these methods since there are usually not so many years of data for many cultivars, especially for new releases from breeding programs (Fadón et al., 2020a). In addition, these approaches can estimate average chill and heat periods, but cannot provide information about dormancy dynamics in a particular year.

The limitations of the currently available methods, the increasing number of new releases in many fruit tree crops, as well as the decrease in winter chilling due to global warming have led to a number of recent research efforts focused on identifying suitable biomarkers to determine the transition from endo- to ecodormancy (Martínez-Gómez et al., 2017; Lloret et al., 2018). These include starch accumulation within the ovary primordia cells (Fadón et al., 2018a) and hormone regulation (Chmielewski et al., 2017; Vimont et al., 2019; Guillamón et al., 2020), as well as the expression of the *DORMANCY-ASSOCIATED MAD-BOX* (DAM) and other candidate genes in several *Prunus* species (Bielenberg et al., 2004; Sánchez-Pérez et al., 2014; Rothkegel et al., 2017; Prudencio et al., 2018, 2019; Balogh et al., 2019; Falavigna et al., 2019; Vimont et al., 2019; Quesada-Traver et al., 2020; Lloret et al., 2021). To deepen insights into the genetics of dormancy, transcriptomic studies have been performed by using flower buds at different dormant stages in apricot (Yu et al., 2020; Canton et al., 2021), sweet cherry (Vimont et al., 2019; Canton et al., 2021), almond (Prudencio et al., 2020), or peach (Yu et al., 2020; Canton et al., 2021). Genes involved in stress response, sugar metabolism, and cell wall assembly contribute to the endodormancy to ecodormancy transition in apricot. Hormone biosynthesis, including ABA catabolism and secondary metabolism, as well as floral and pollen development genes are relevant at ecodormancy (Yu et al., 2020). Sporophytic genes, such as *MALE STERILE1* (*MS1*), *ABORTED MICROSPORES* (*AMS*), and *MYB103* (Canton et al., 2021), as well as other genes involved in sporopollenin synthesis and deposition found in peach (Ríos et al., 2013), could be useful markers of the ecodormancy stage.

In apricot, the characterization of the phases of pollen development in relation to dormancy established that male meiosis is the first detectable sign of development after endodormancy breaking (Julian et al., 2011), when flower buds are still closed with no observable external phenological changes (Fadón et al., 2018b). This led us to use male meiosis as a biomarker to estimate the AR of cultivars for the first time by using the emergence of callose around the microspore mother cells as a boundary between endo- and ecodormancy. Callose deposition can be considered as a physical filter of molecules that have been reported as a response to several stresses (Chen and Kim, 2009) or degeneration processes, such as ovule abortion (Rodrigo and Herrero, 1998). During pollen development, the accumulation of a layer of callose around the microspore mother cells could provide insulation and possible protection against low winter temperatures.

Since the statistical analyses showed good agreement when male meiosis was compared to the other methods, these results strongly endorse the use of male meiosis as a new and alternative method to determine endodormancy release in apricot. The results obtained by this approach showed slightly higher values than those obtained by the other methods because male meiosis occurs in the early stages of ecodormancy, once the endodormancy breaking had already passed. Despite this limitation, this method has an important advantage since it allowed the determination of endo- and ecodormancy in a short time from samples collected directly from the field, avoiding the variability of results caused by the many factors that may affect the experiments under forced conditions (Dennis, 2003; Fadón and Rodrigo, 2018) and without the need to have a large phenological data set available (Luedeling and Gassner, 2012; Fadón et al., 2020a). Despite the important advantages of this method in estimating CR in apricot, endodormancy breaking and male meiosis are not as closely related processes in other species as sweet cherry, in which several months elapse between both processes (Fadón et al., 2019) and this could lead to overestimated calculations of AR (Fadón et al., 2021). This may be because sweet cherry requires a longer forcing period to flower (Fadón et al., 2020b). However, male meiosis as a biomarker of endodormancy could be an applicable method in those species with a shorter forcing phase in which flowering is more conditioned by the chilling phase, such as almond (Egea et al., 2003) or Japanese plum (Ruiz et al., 2018). Although further studies are needed to determine if this method is valid in other species, our results exhibit male meiosis as a valid approach for apricot, not only for determining AR but also as a basis for physiological and genetic studies of dormancy. In addition, whether a bud is an endo- or ecodormant can be determined in a short time from a sample taken directly from the tree in field conditions. The knowledge of the endo- to ecodormancy transition may provide valuable information to improve and simplify orchard management decisions. The progressive loss of cold hardiness is triggered once the buds have fulfilled their CR (Weiser, 1970a,b) but show no external signs of development (Julian et al., 2007; Hillmann et al., 2021). Determining endodormancy breaking in apricot through the detection of male meiosis could provide insights into whether flower buds are vulnerable to spring frosts, allowing agronomic frost protection decisions to be made. In addition, the date of male meiosis could allow the determination of the optimal application time of agrochemical treatments to advance flowering (Guillamón et al., 2022).

## Data Availability Statement

The original contributions presented in the study are included in the article/Supplementary Material, further inquiries can be directed to the corresponding author/s.

## Author Contributions

SH, JH, JL, and JR conceived the study and designed the experiments. SH collected the shoot and bud samples and performed the microscopic analysis. SH and JA performed the correlation analysis. SH and EF performed the PLS analysis. SH and AH performed the statistical analysis. SH, JL, EF, AH, JA, JH, and JR analyzed the results and wrote the manuscript. All authors contributed to the article and approved the submitted version.

## Conflict of Interest

The authors declare that the research was conducted in the absence of any commercial or financial relationships that could be construed as a potential conflict of interest.

## Publisher’s Note

All claims expressed in this article are solely those of the authors and do not necessarily represent those of their affiliated organizations, or those of the publisher, the editors and the reviewers. Any product that may be evaluated in this article, or claim that may be made by its manufacturer, is not guaranteed or endorsed by the publisher.

## Supplementary Material

Supplementary Figure 1 Establishment of endodormancy breaking date (T) in 20 apricot cultivars by analyzing the evolution of the Pearson correlation coefficients between full flowering dates and winter temperatures of periods of 15 days. Discontinuous lines delimit the significant values (0.553, –0.553). Green and red scales show positive and negative coefficients, respectively.

Supplementary Figure 2 Model coefficients of Partial Least Squares regression between accumulation rates of agroclimatic metrics (Chill Portions according to the Dynamic Model and Growing Degree Hours) and bloom dates of 20 apricot cultivars. Top panels show the Variable importance in the projection (VIP), with the blue bars values above 0.8 indicating the threshold for variable importance. Middle panels show the model coefficients of the centered and scaled data. Chilling period is colored in blue and heating period is colored in red. Bottom panels show mean temperatures (black line) and their standard deviation (grey areas). In middle and bottom panels, red and green scales show negative and positive coefficients, respectively.

Supplementary Table 1 Chilling and heat requirements of 20 apricot cultivars determined by the Forcing test. Dates of endodormancy and full flowering, yearly values of chill accumulation expressed in chill portions, chill units, and chill hours below 7°C, yearly values of heat accumulation expressed in Growing Degree Hours (GDH), mean values, standard deviation (SD), and coefficient of variation (cv).

Supplementary Table 2 Chilling and heat requirements of 20 apricot cultivars determined by the Correlation method. Dates of endodormancy and full flowering, values of chill accumulation expressed in chill portions, chill units, and hours below 7°C, values of heat accumulation expressed in Growing Degree Hours (GDH), standard deviation (SD), and coefficient of variation (cv).

Supplementary Table 3 Chilling and heating requirements of 20 apricot cultivars determined by the PLS regression method. Chilling and forcing periods are established with Partial Least Squares regression based on the Dynamic Model and the Growing Degree Hours Model. Chill accumulation is expressed in chill portions, chill units, and hours below 7°C, values of heat accumulation are expressed in Growing Degree Hours (GDH), standard deviation (SD), and coefficient of variation (cv).

Supplementary Table 4 Chilling and heating requirements of 20 apricot cultivars determined by the Male meiosis method. Dates of endodormancy and full flowering, yearly values of chill accumulation expressed in chill portions, chill units, and hours below 7°C, yearly values of heat accumulation expressed in Growing Degree Hours (GDH), mean values, standard deviation (SD), and coefficient of variation (cv).
