# Supplementary material for: Male Meiosis as a Biomarker for Endo- to Ecodormancy Transition in Apricot
Source: Front Plant Sci. 2022 Apr 7;13:842333. doi: 10.3389/fpls.2022.842333 (PMC9021868; doi:10.3389/fpls.2022.842333)
Supplement: Supplementary Figure 1 — Establishment of endodormancy breaking date (T) in 20 apricot cultivars by analyzing the evolution of the Pearson correlation coefficients between full flowering dates and winter temperatures of periods of 15 days. Discontinuous lines delimit the significant values (0.553, –0.553). Green and red scales show positive and negative coefficients, respectively. [file Image_1.pdf]

Berdejo

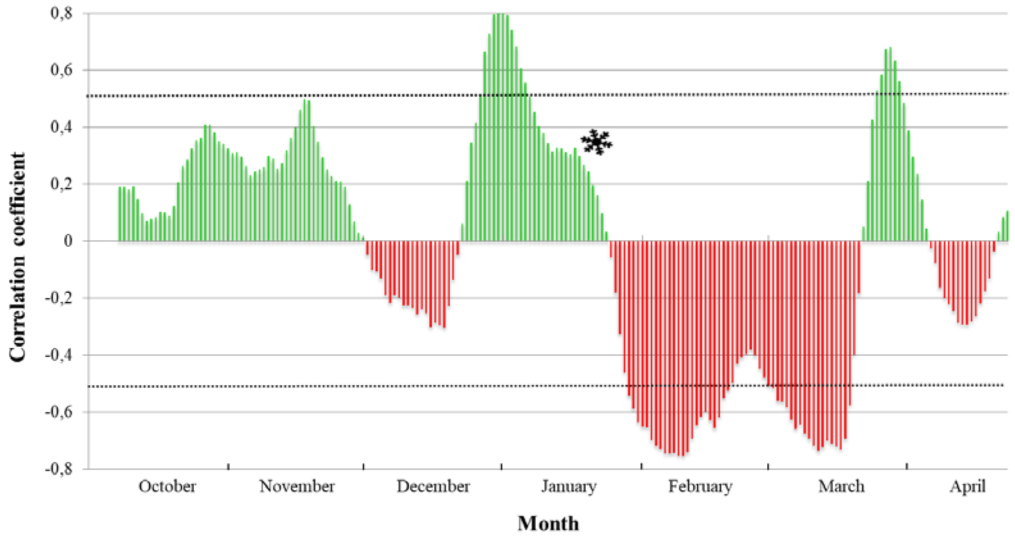

Canino

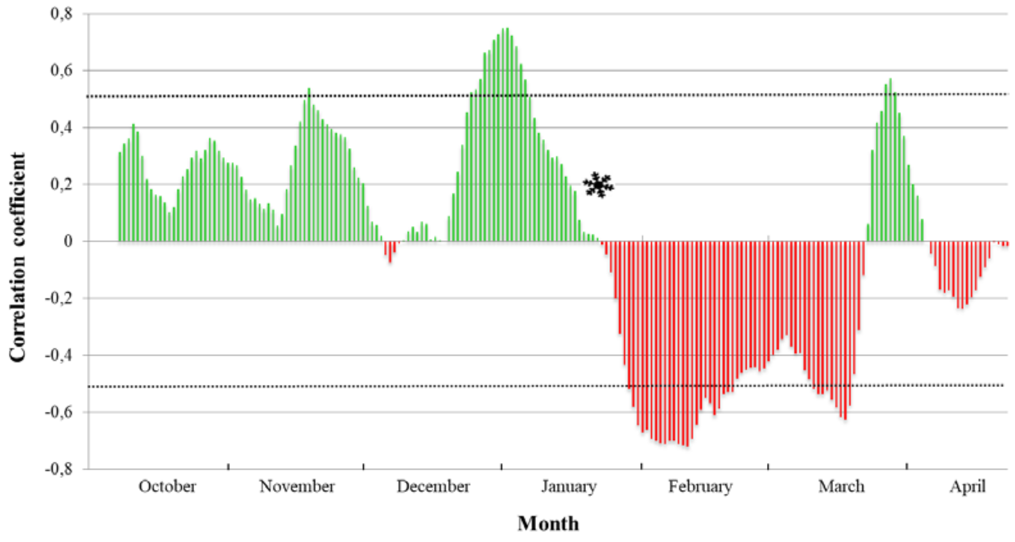

Corbato

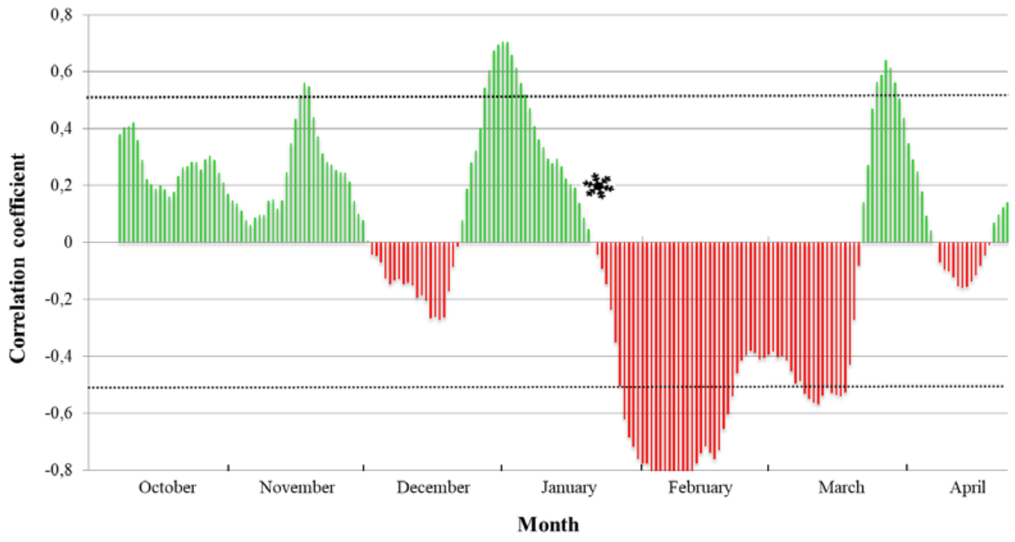

Goldrich

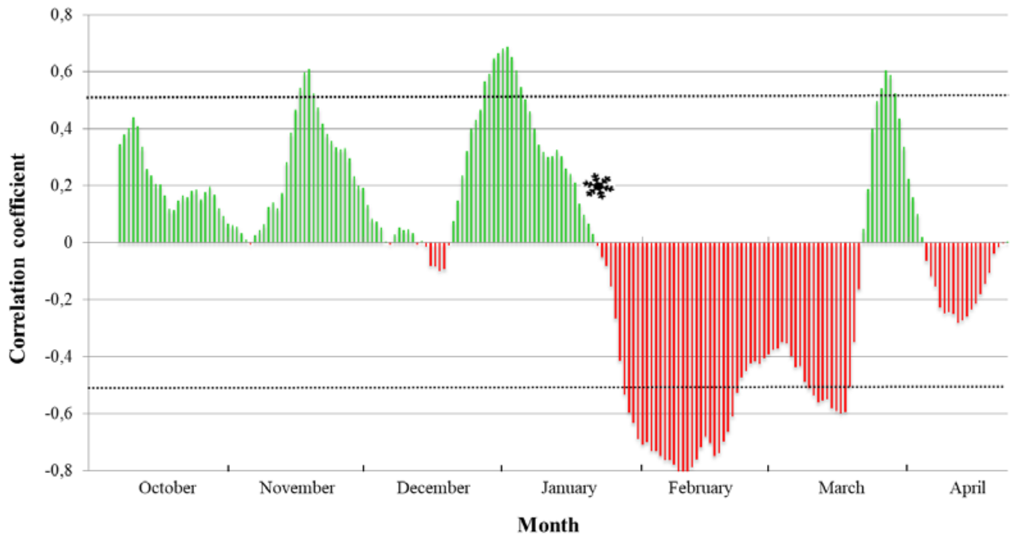

Gonci Magiar

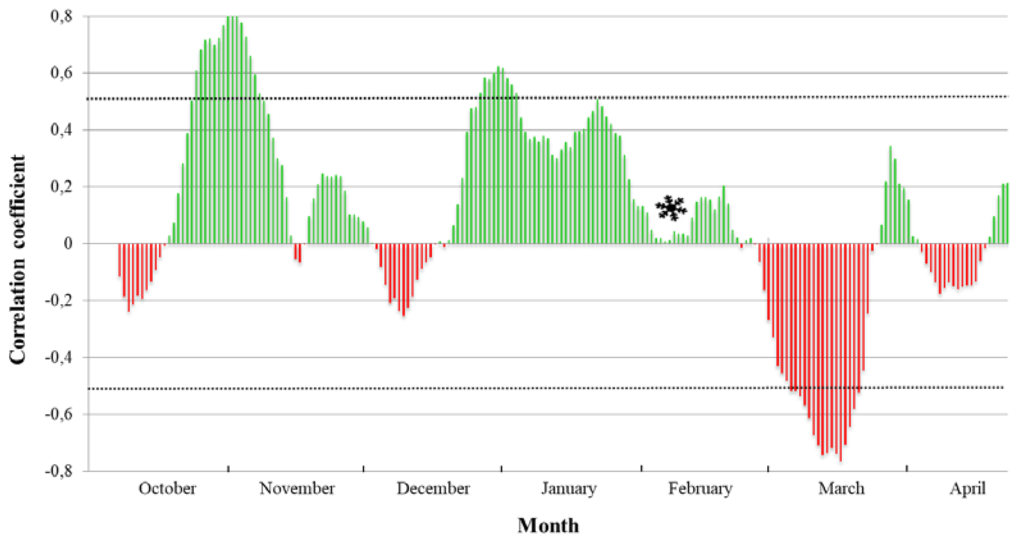

Harcot

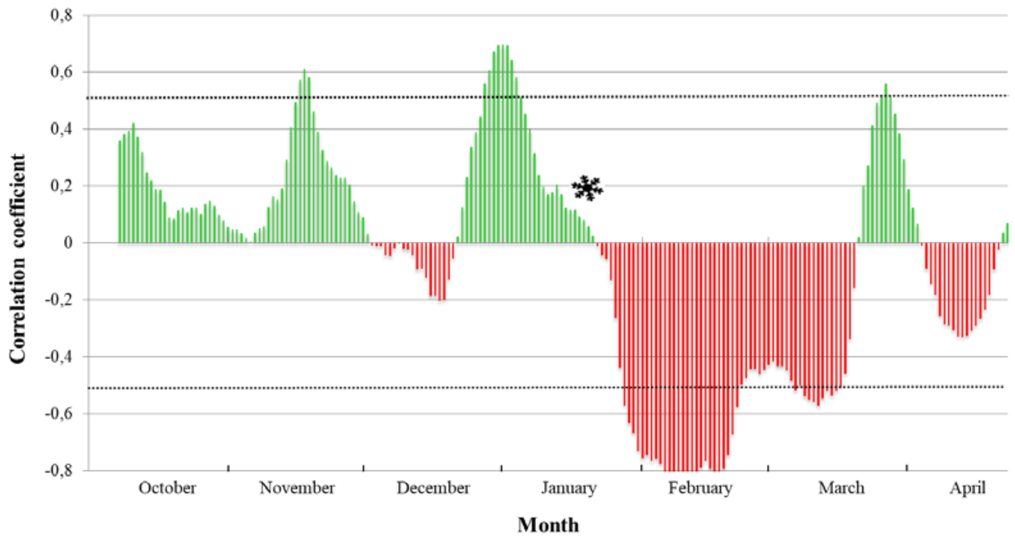

Henderson

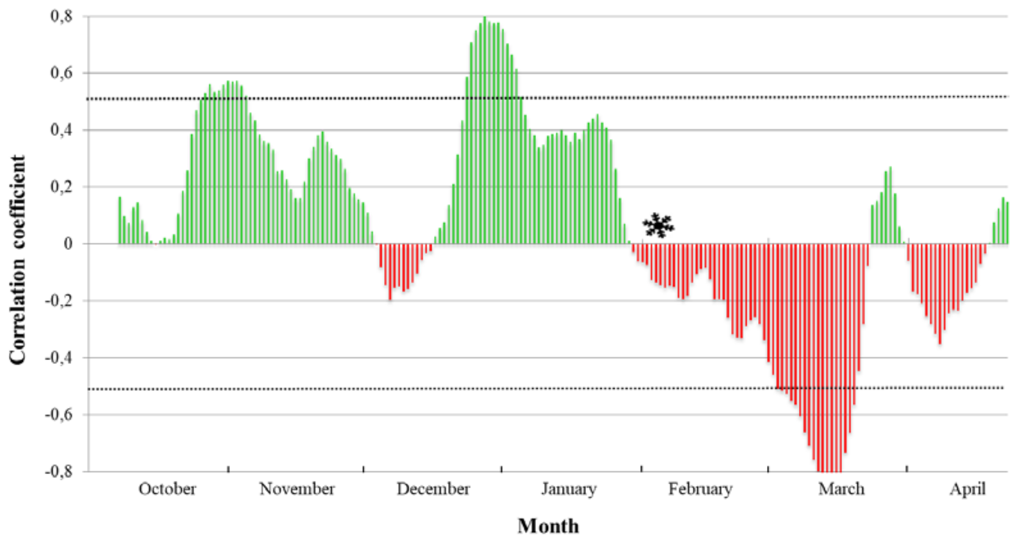

Luizet

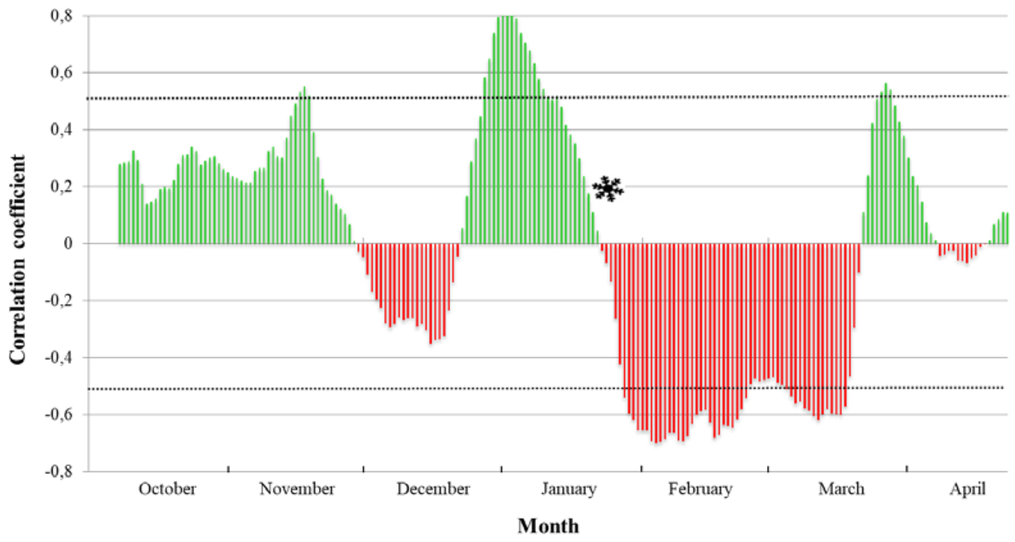

Mitger

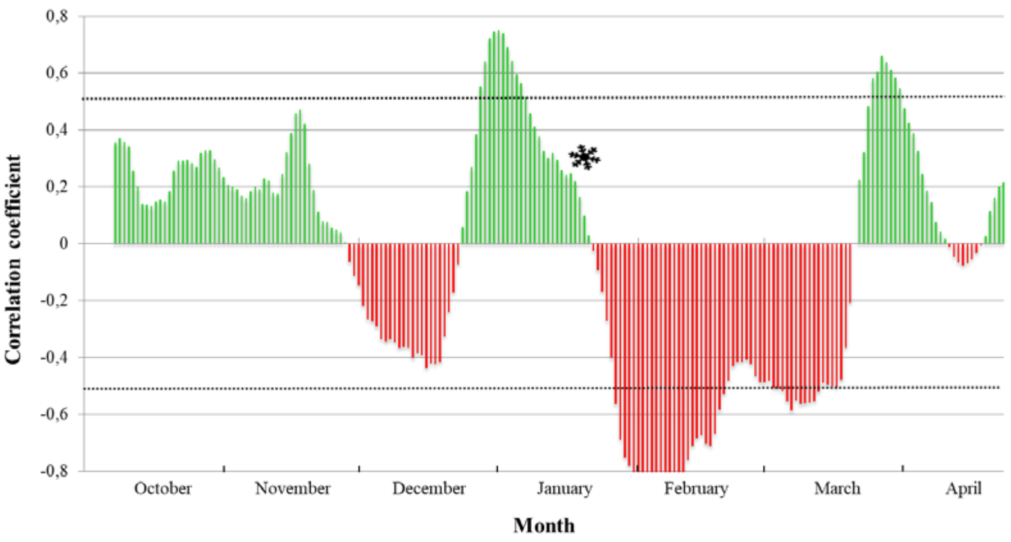

Moniqui 1006

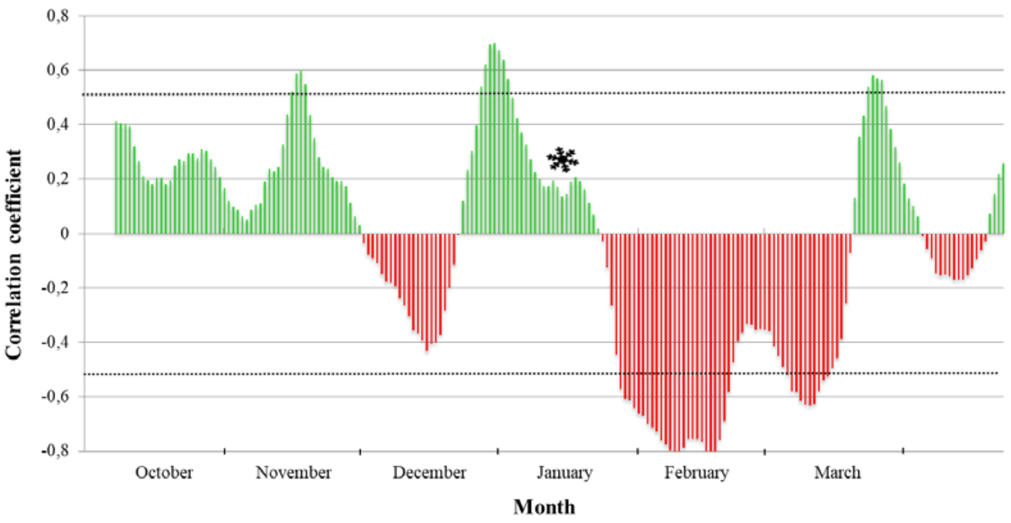

Moniqui 2113

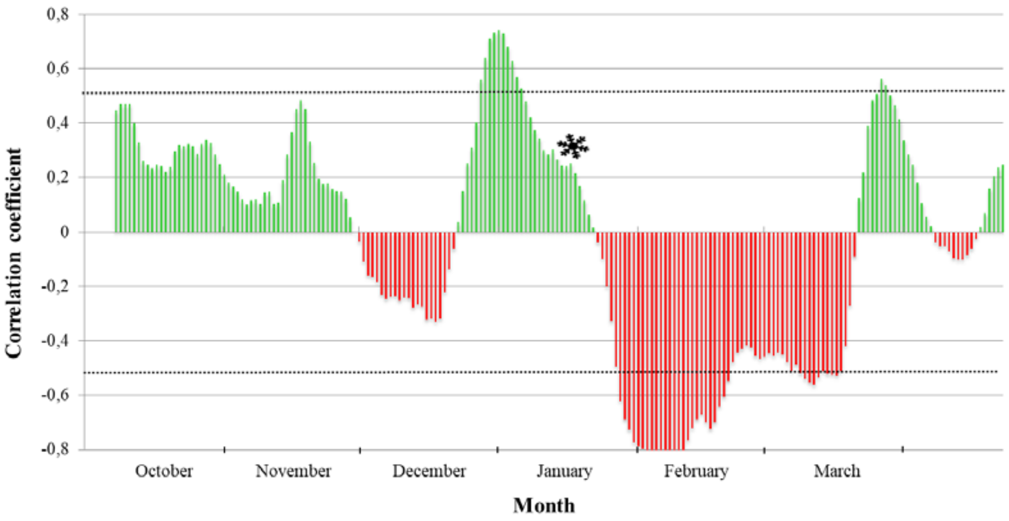

Muñoz

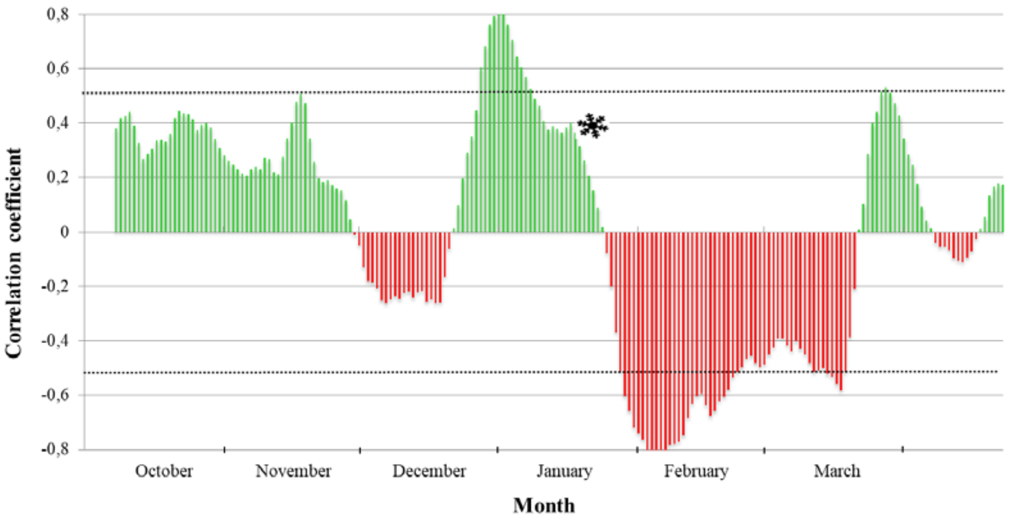

Pandora

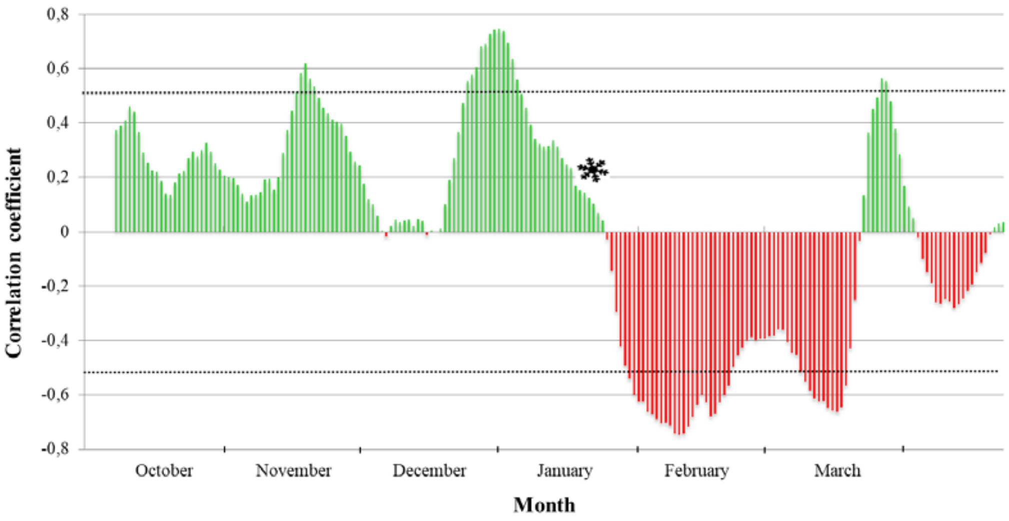

Paviot

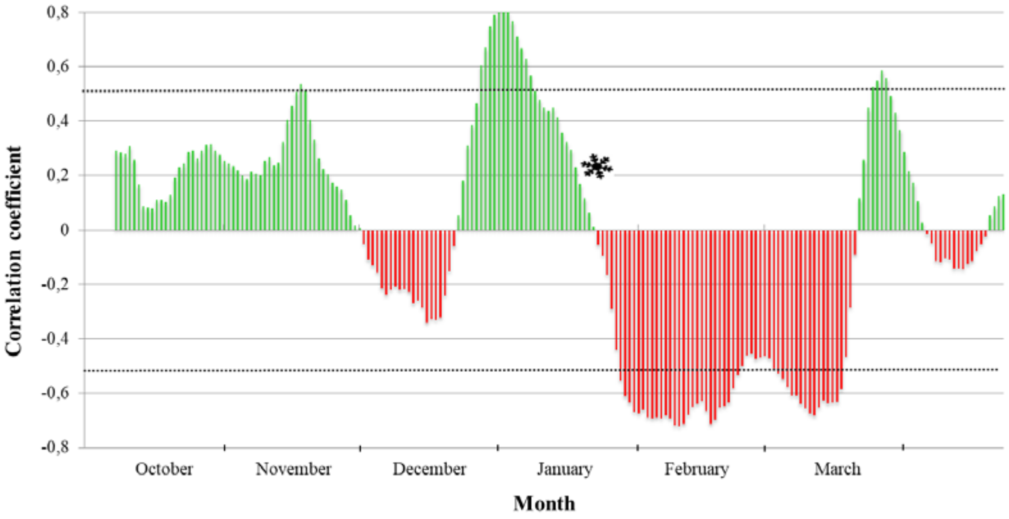

Pepito del Rubio

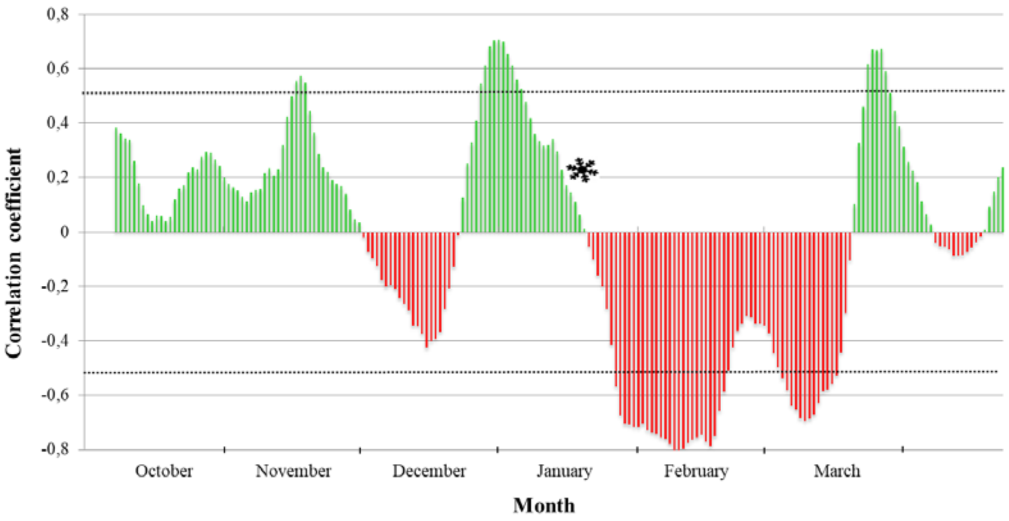

Stark Early Orange

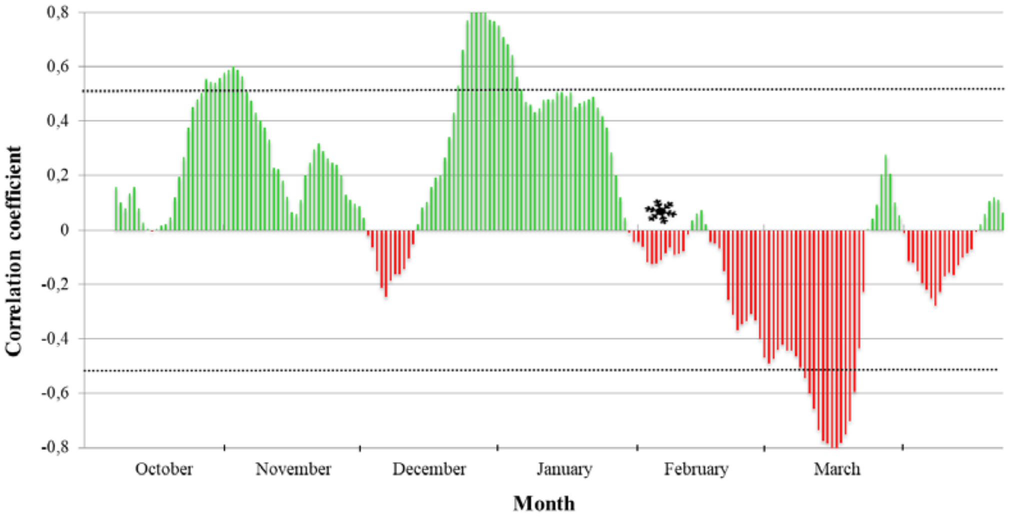

Stella

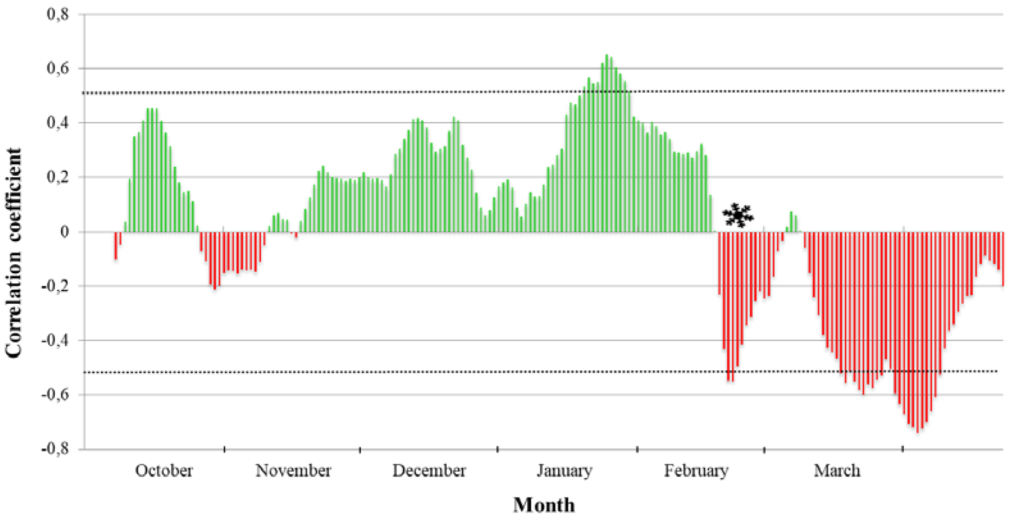

Sun Glo

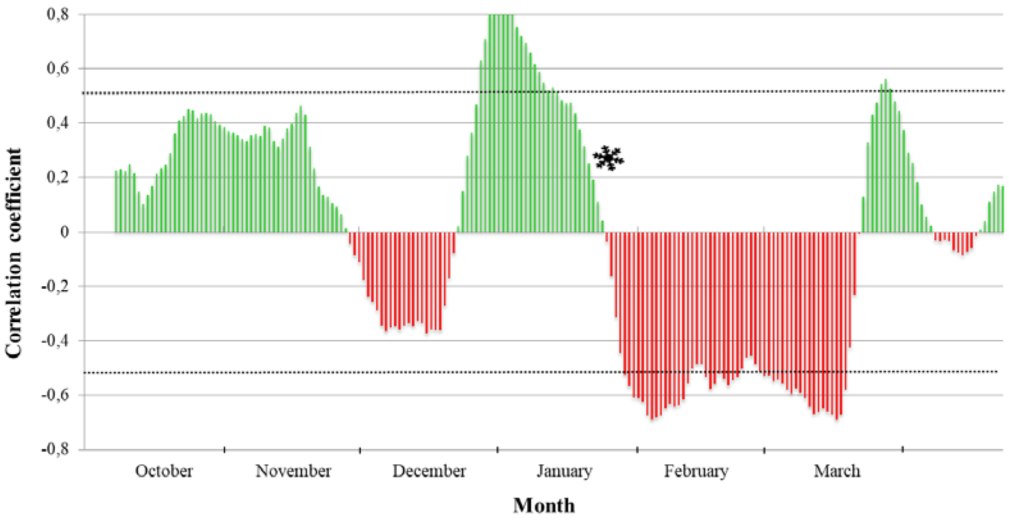

Tadeo

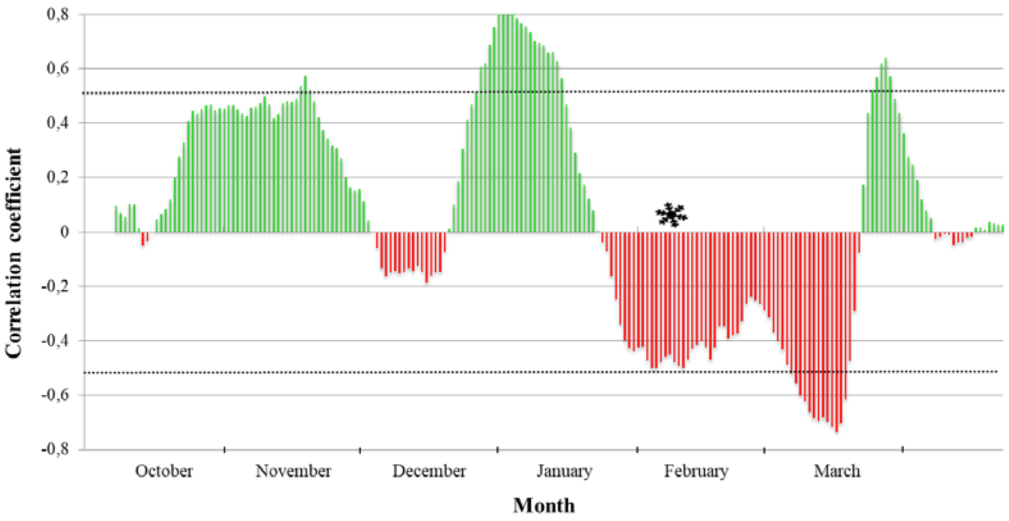

Veecot

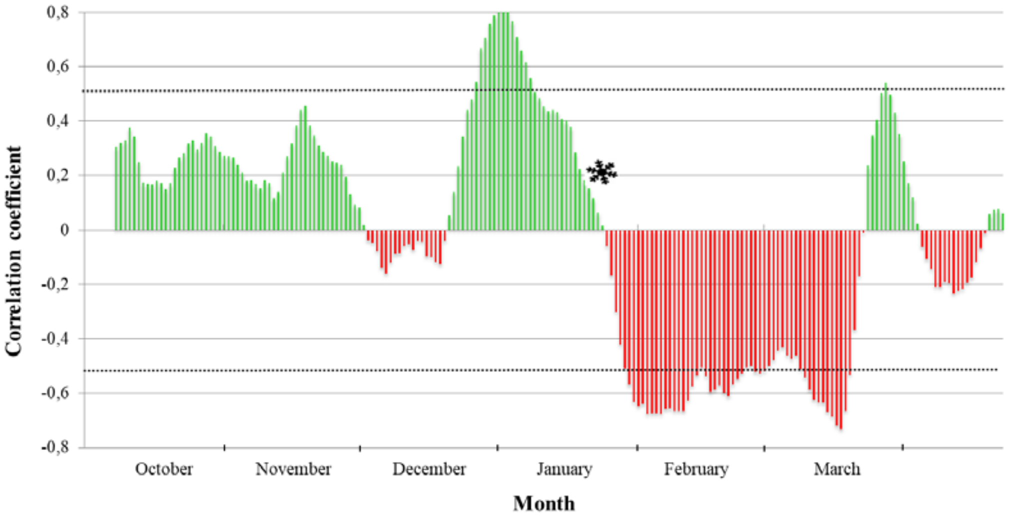

Supplementary Figure S1. Establishment of endodormancy breaking date (❄) in 20 apricot cultivars by analyzing the evolution of the Pearson correlation coefficients between full flowering dates and winter temperatures of periods of 15 days. Discontinuous lines delimit the significant values (0.553, -0.553). Green and red scales show positive and negative coefficients, respectively.
