# Supplementary material for: Male Meiosis as a Biomarker for Endo- to Ecodormancy Transition in Apricot
Source: Front Plant Sci. 2022 Apr 7;13:842333. doi: 10.3389/fpls.2022.842333 (PMC9021868; doi:10.3389/fpls.2022.842333)
Supplement: Supplementary Figure 2 — Model coefficients of Partial Least Squares regression between accumulation rates of agroclimatic metrics (Chill Portions according to the Dynamic Model and Growing Degree Hours) and bloom dates of 20 apricot cultivars. Top panels show the Variable importance in the projection (VIP), with the blue bars values above 0.8 indicating the threshold for variable importance. Middle panels show the model coefficients of the centered and scaled data. Chilling period is colored in blue and heating period is colored in red. Bottom panels show mean temperatures (black line) and their standard deviation (grey areas). In middle and bottom panels, red and green scales show negative and positive coefficients, respectively. [file Image_2.pdf]

Berdejo

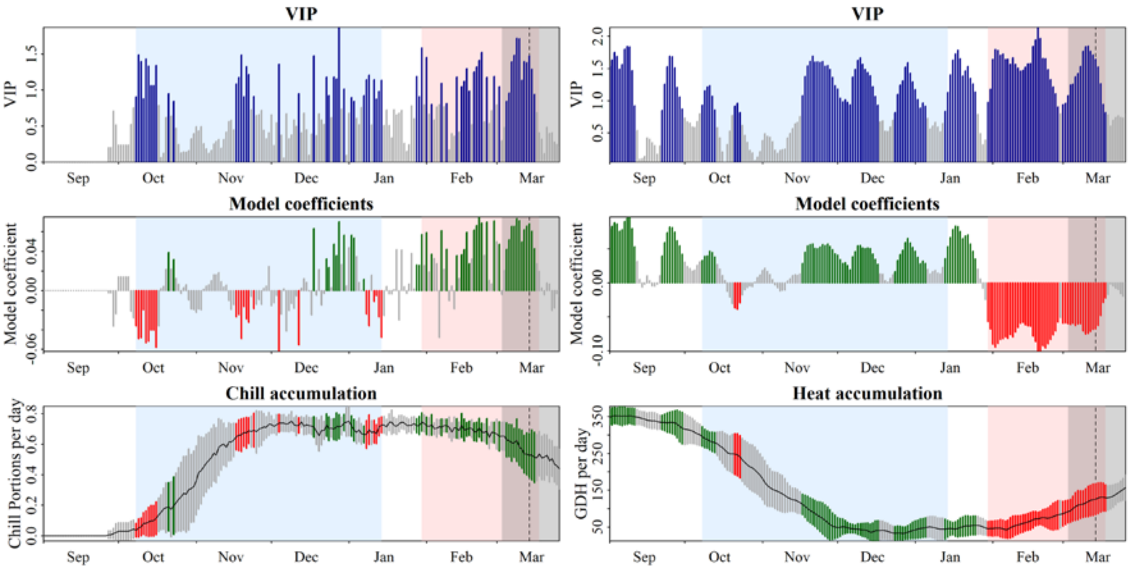

Canino

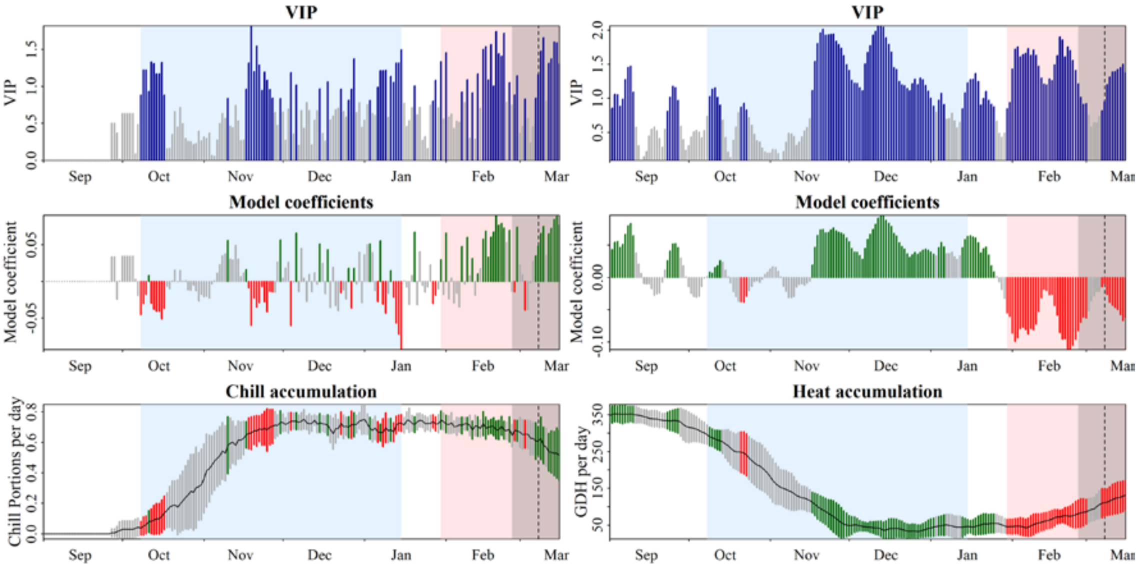

Corbato

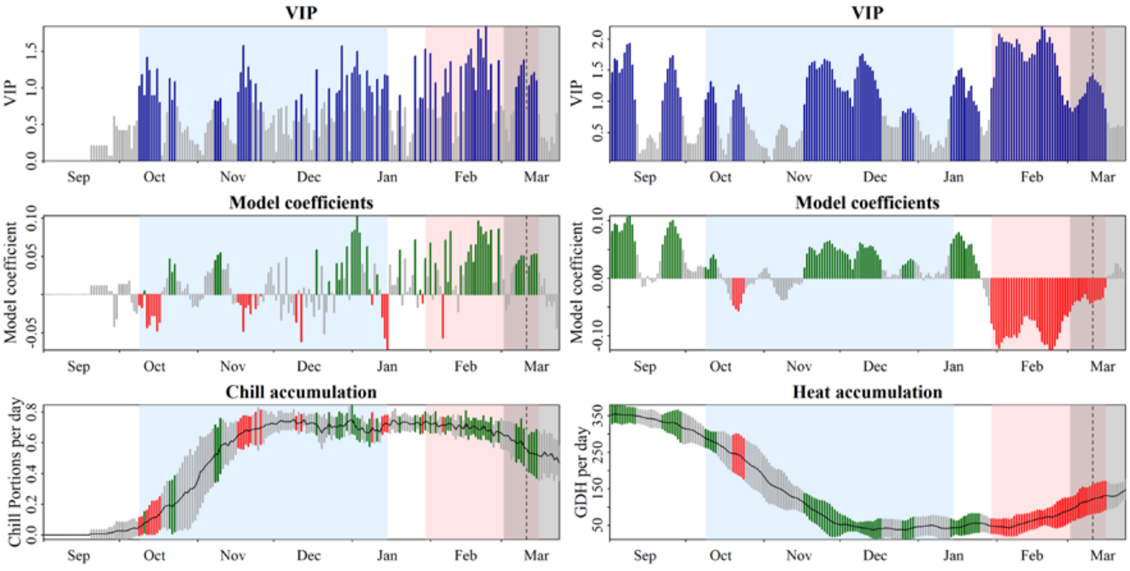

Goldrich

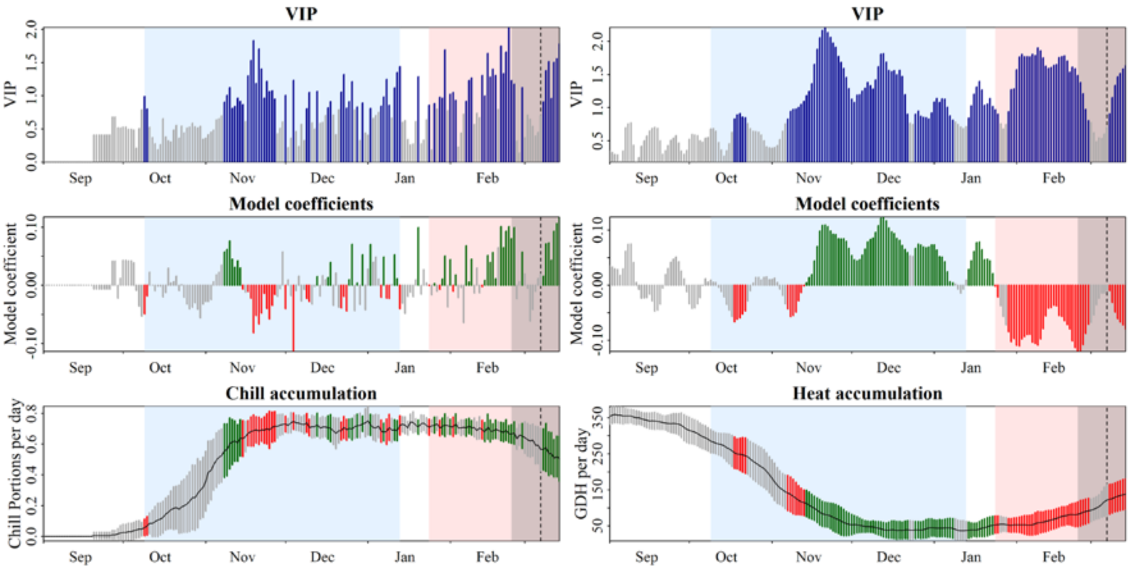

Gonci Magiar

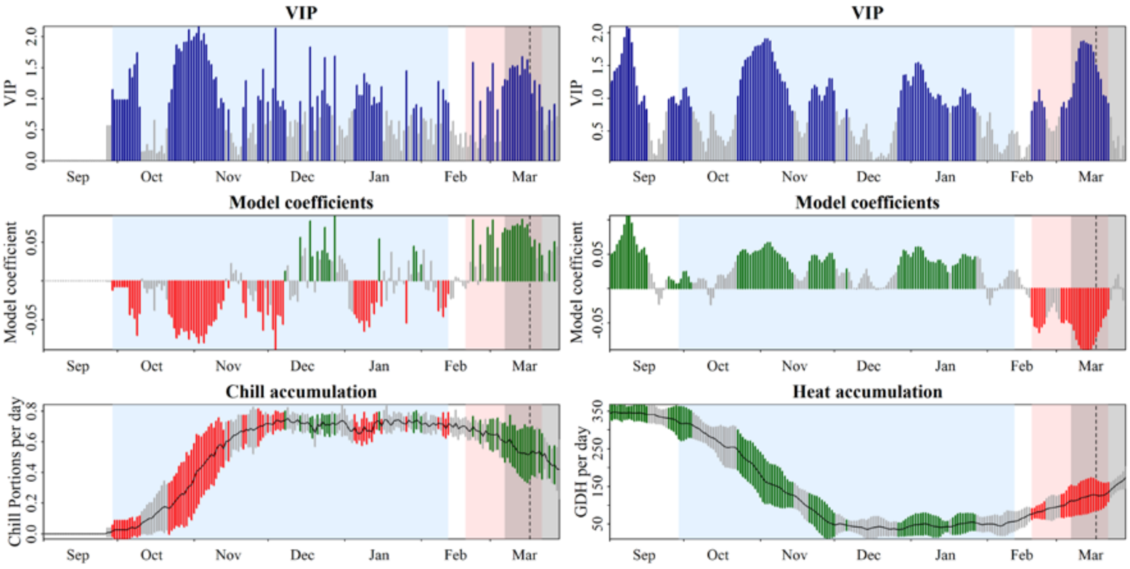

Harcot

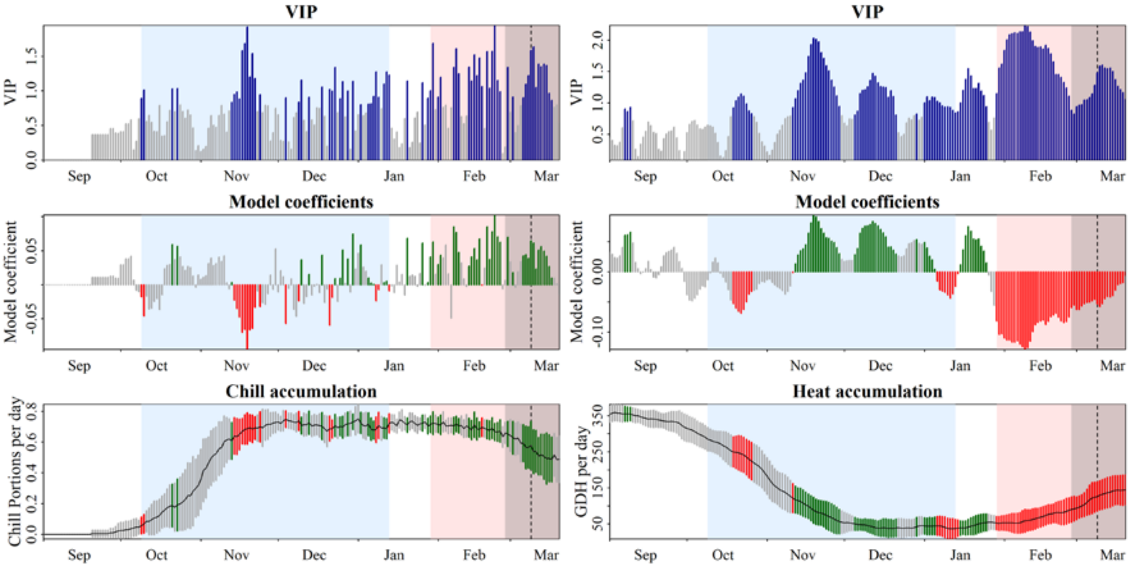

Henderson

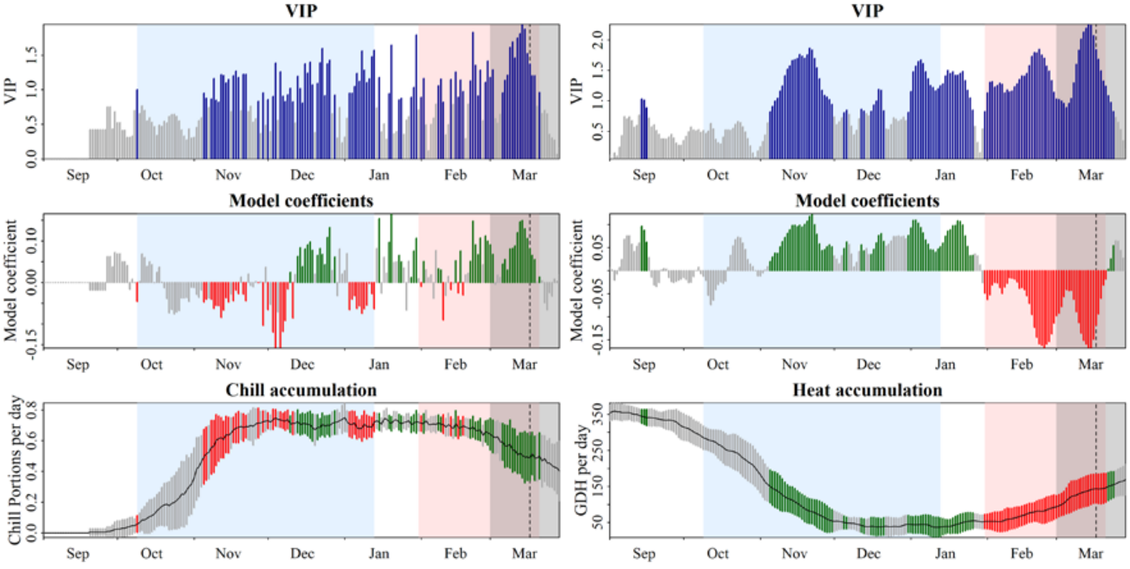

Luizet

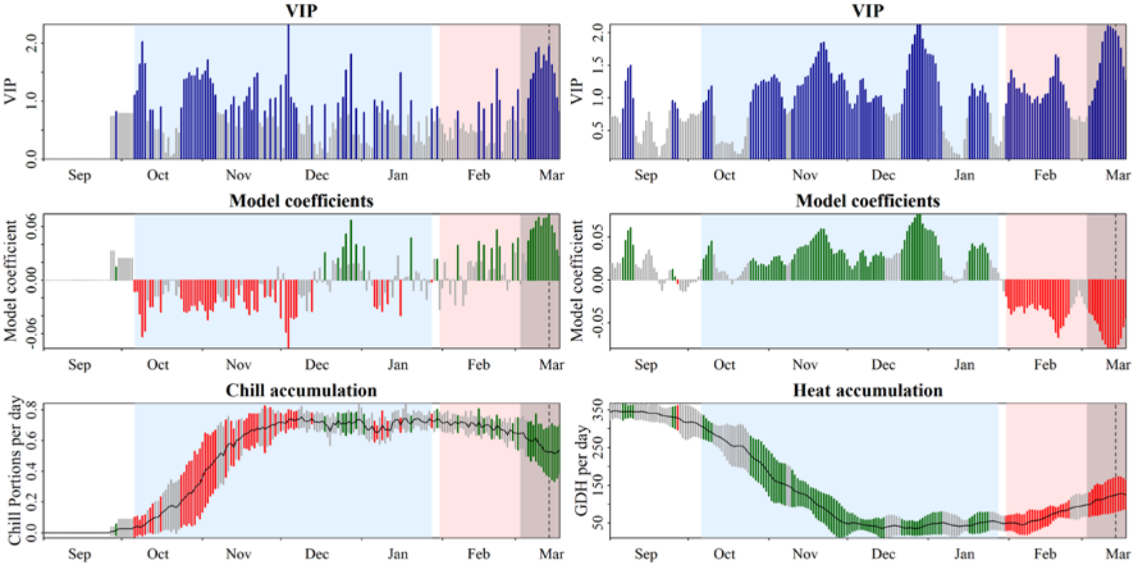

Mitger

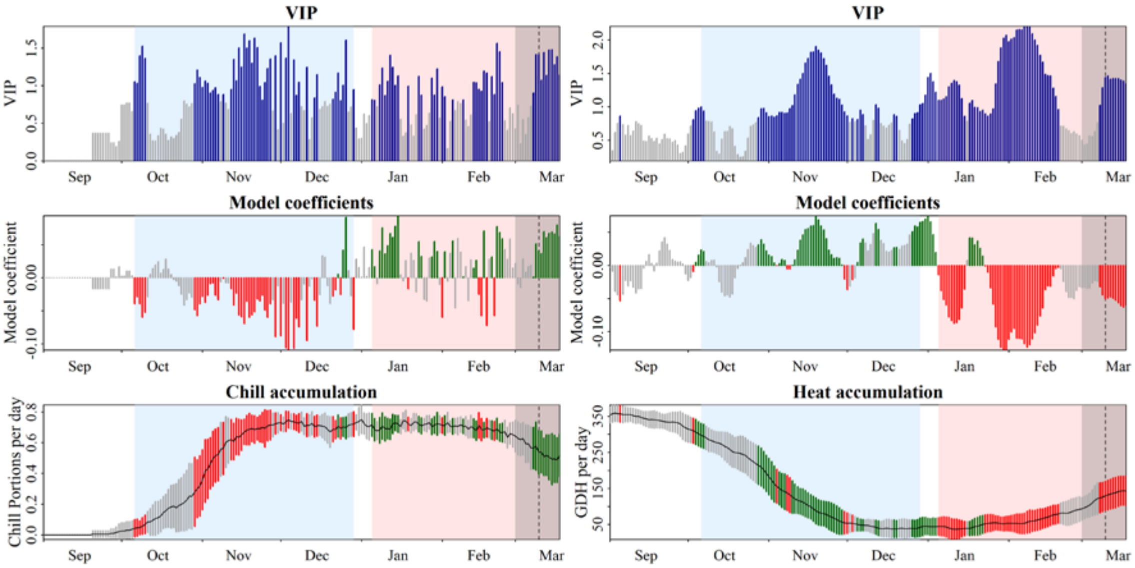

Moniqui 1006

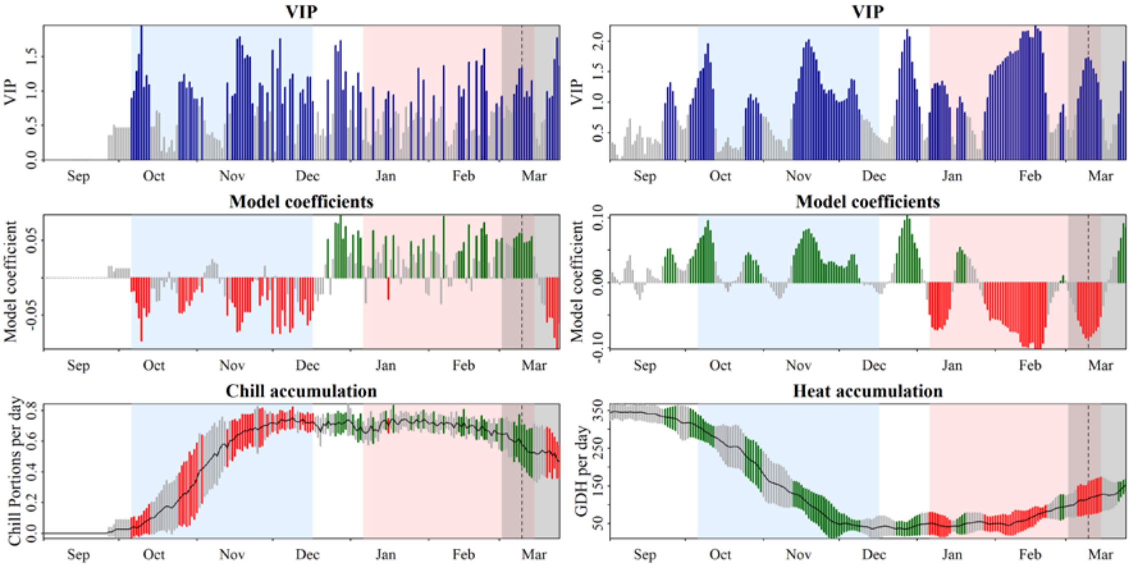

Moniqui 2113

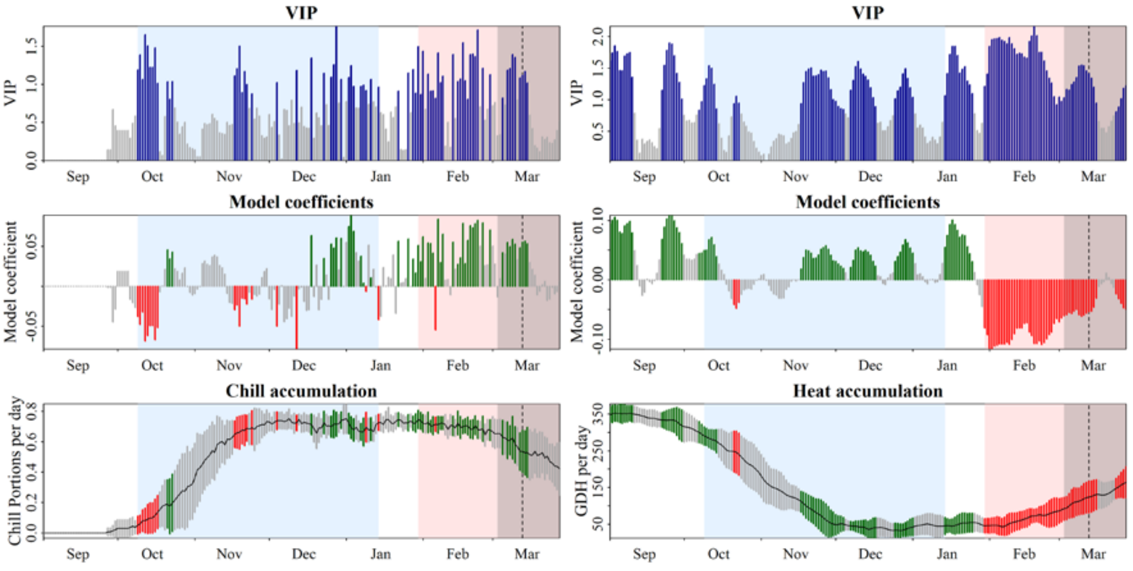

Muñoz

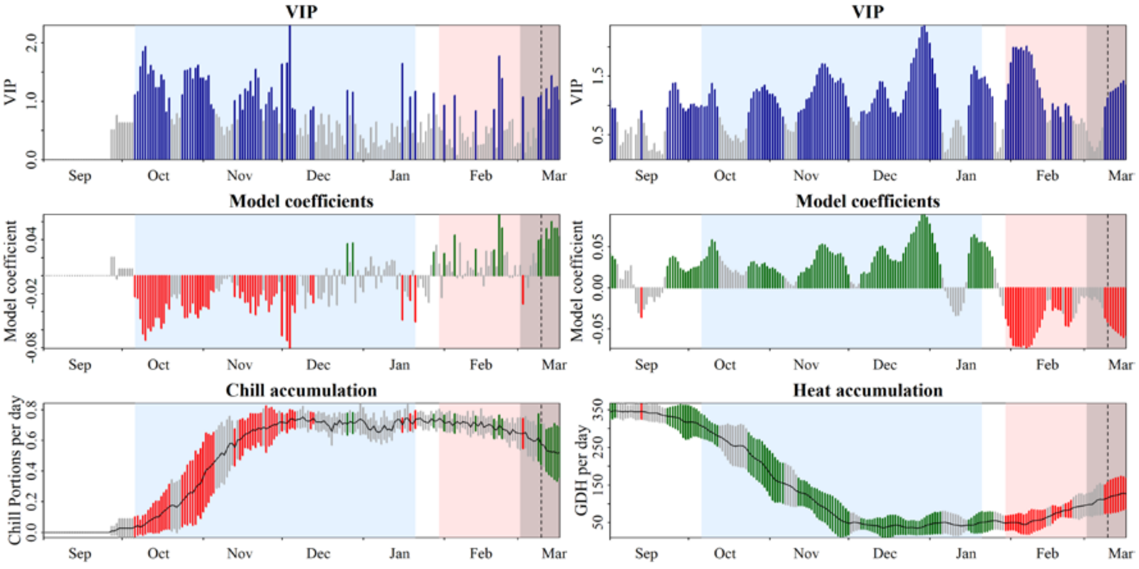

Pandora

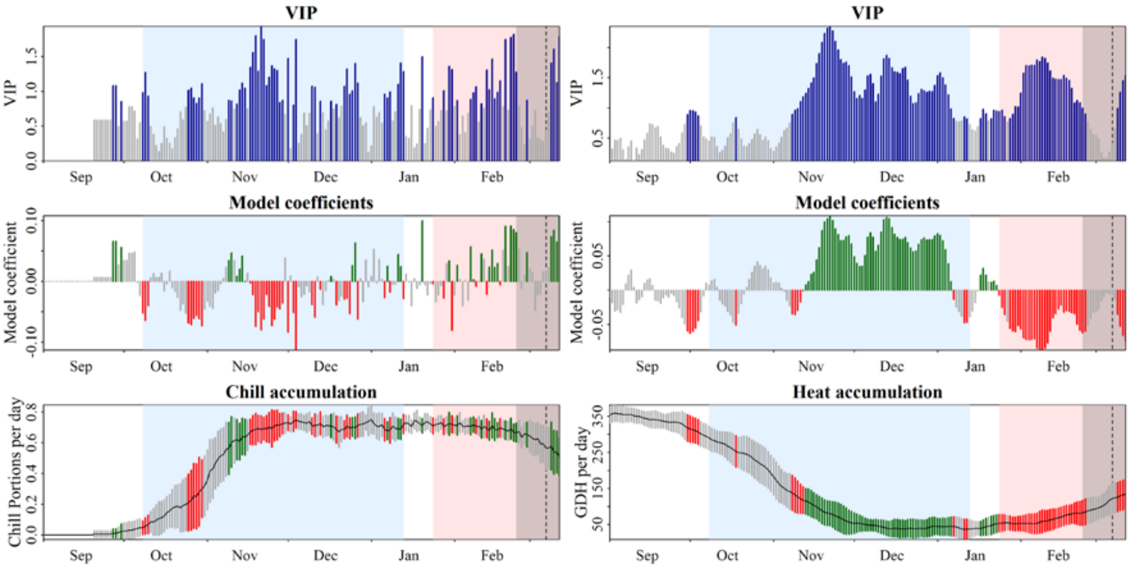

Paviot

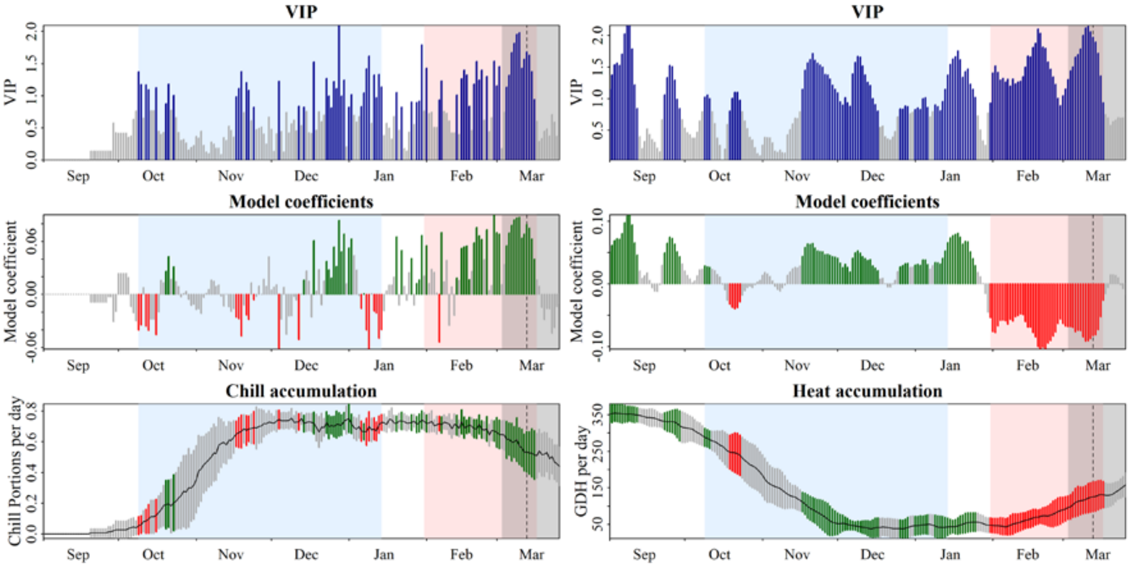

Pepito del Rubio

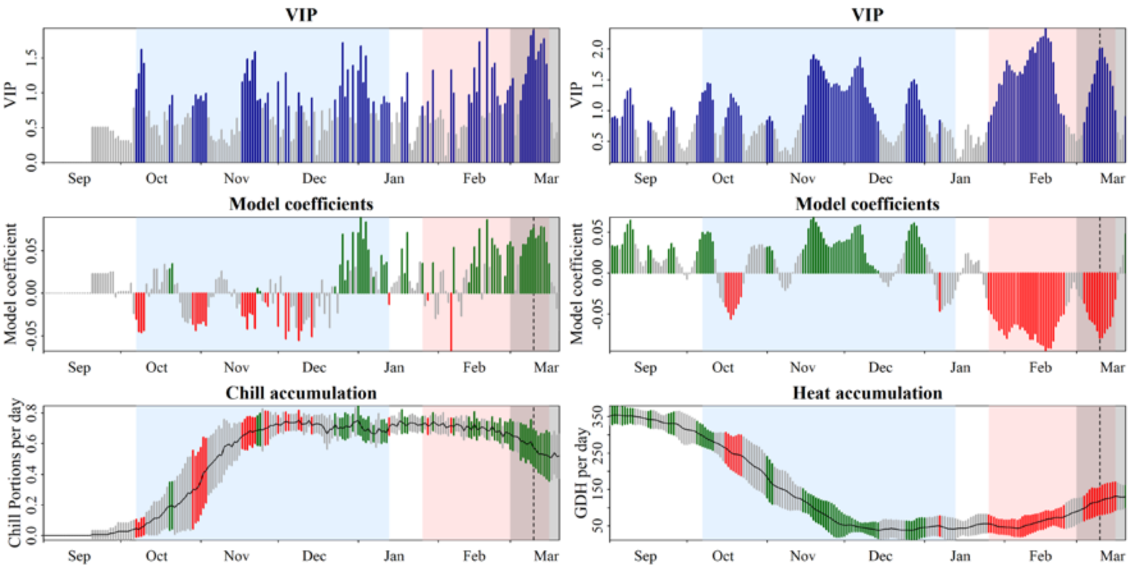

Stark Early Orange

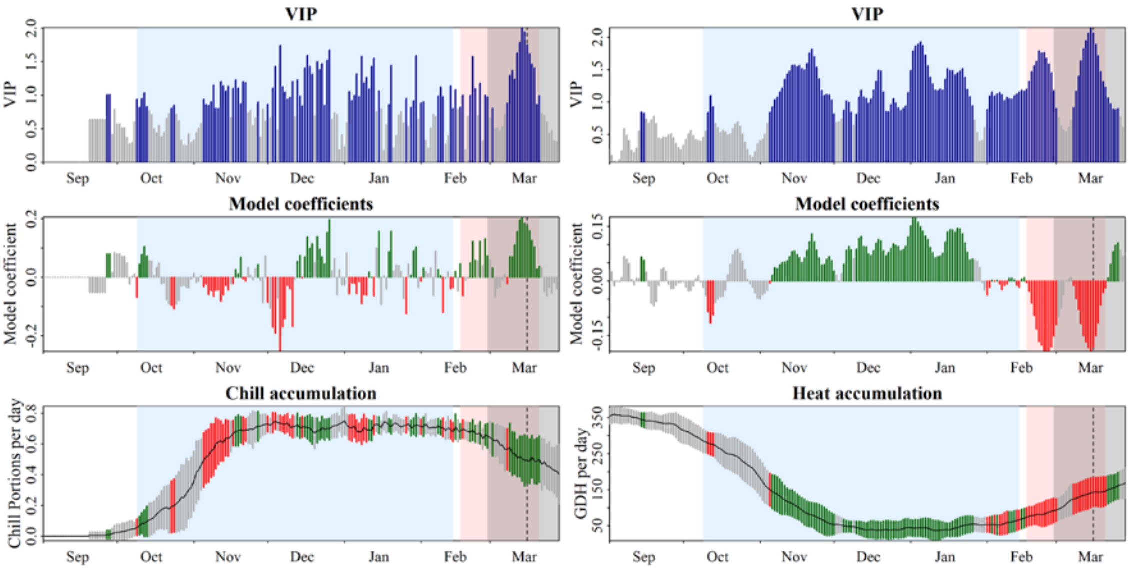

Stella

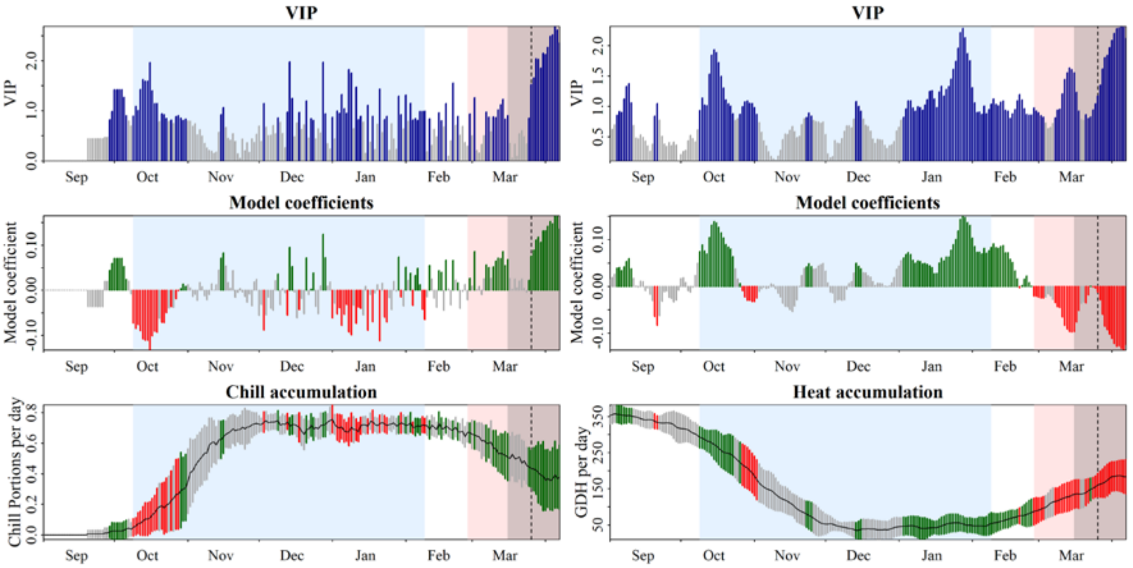

Sun Glo

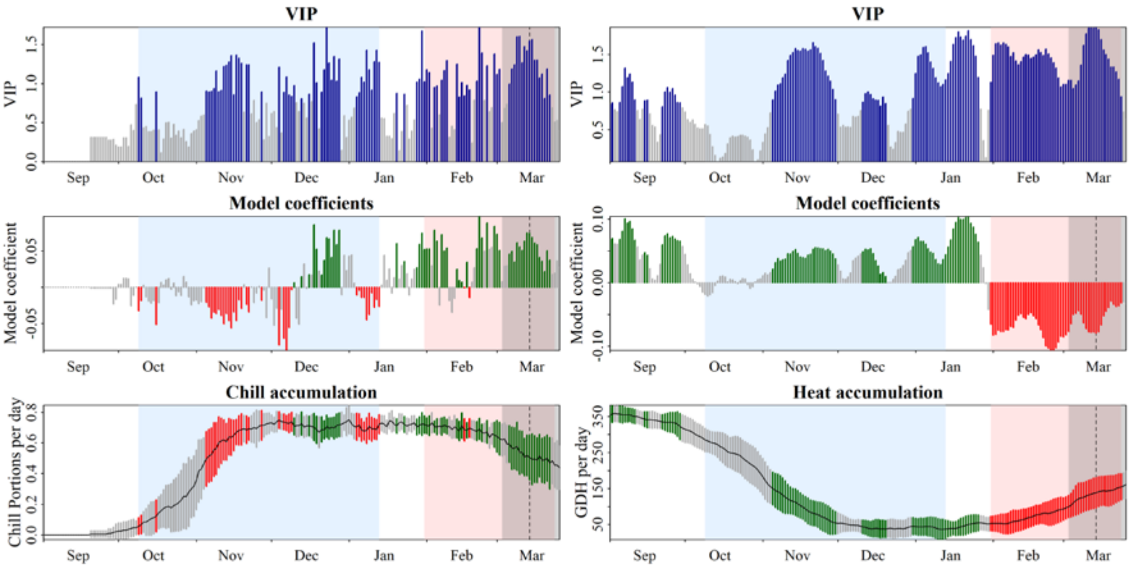

Tadeo

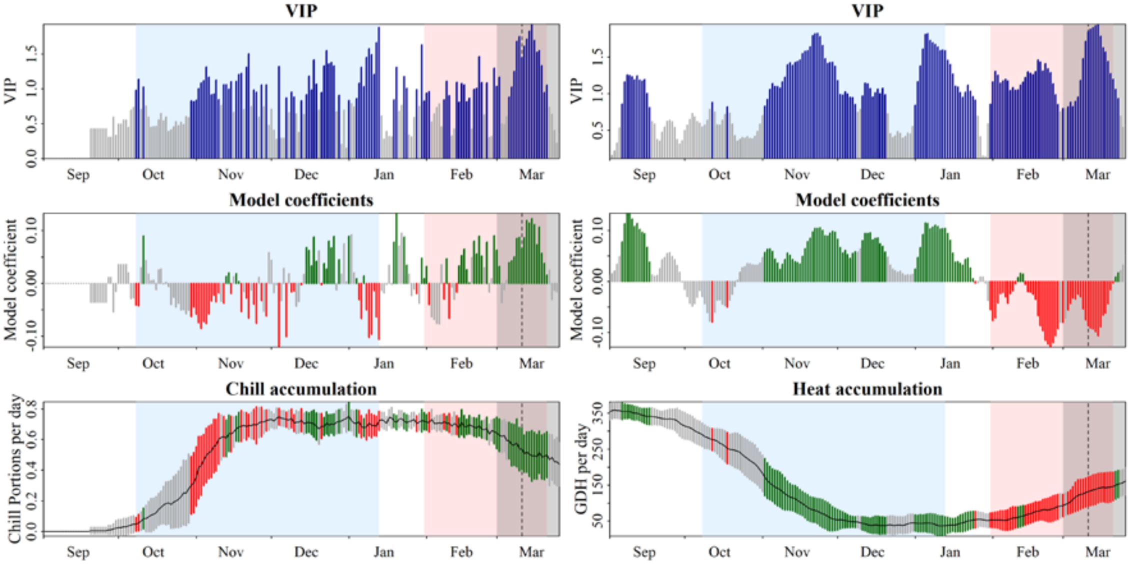

Veecot

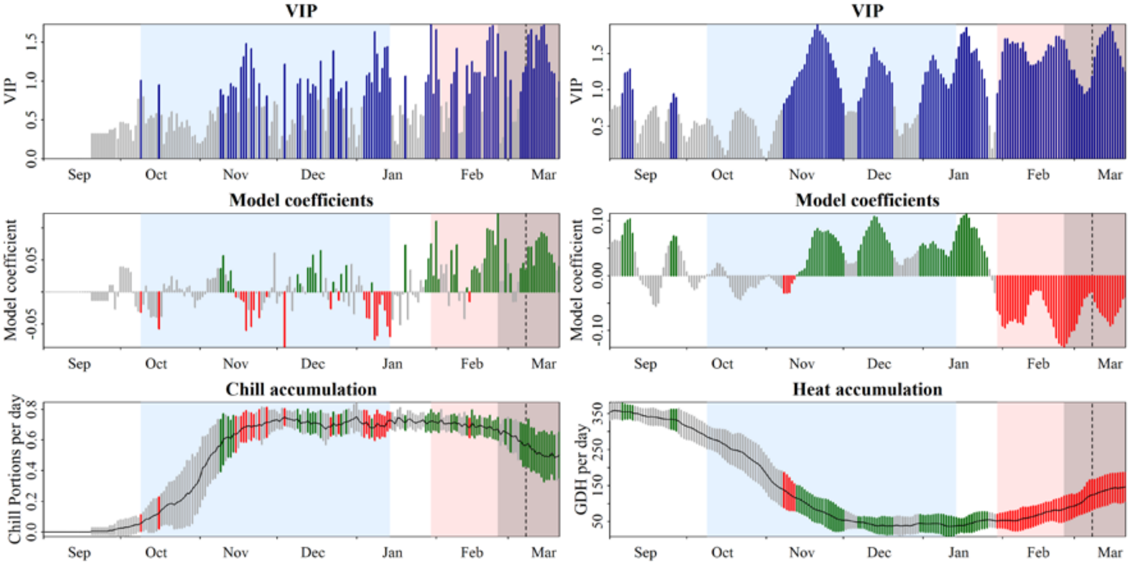

Supplementary Figure S2. Supplementary Figure S2. Model coefficients of Partial Least Squares regression between accumulation rates of agroclimatic metrics (Chill Portions according to the Dynamic Model and Growing Degree Hours) and bloom dates of 20 apricot cultivars. Top panels show the Variable importance in the projection (VIP), with the blue bars values above 0.8 indicating the threshold for variable importance. Middle panels show the model coefficients of the centered and scaled data. Chilling period is colored in blue and heating period is colored in red. Bottom panels show mean temperatures (black line) and their standard deviation (grey areas). In middle and bottom panels, red and green scales show negative and positive coefficients, respectively
